# Melanin-like polydopamine nanoparticles mediating anti-inflammatory and rescuing synaptic loss for inflammatory depression therapy

**DOI:** 10.1186/s12951-023-01807-4

**Published:** 2023-02-10

**Authors:** Ting-ting Zhu, He Wang, Han-wen Gu, Ling-sha Ju, Xin-miao Wu, Wei-tong Pan, Ming-ming Zhao, Jian-jun Yang, Pan-miao Liu

**Affiliations:** 1 Department of Anesthesiology, Pain and Perioperative Medicine, The First Affiliated Hospital of Zhengzhou University, Zhengzhou, 450052 China; 2 Neuroscience Research InstituteZhengzhou University Academy of Medical Sciences, Zhengzhou University, Zhengzhou, 450052 China

**Keywords:** Polydopamine nanoparticles, Lipopolysaccharide, Neuroinflammation, Synaptic loss, Depression

## Abstract

Inflammatory depression is closely related to neuroinflammation. However, current anti-inflammatory drugs have low permeability to cross blood–brain barrier with difficulties reaching the central nervous system to provide therapeutic effectiveness. To overcome this limitation, the nano-based drug delivery technology was used to synthesize melanin-like polydopamine nanoparticles (PDA NPs) (~ 250 nm) which can cross the blood–brain barrier. Importantly, PDA NPs with abundant phenolic hydroxyl groups function as excellent free radical scavengers to attenuate cell damage caused by reactive oxygen species or acute inflammation. In vitro experiments revealed that PDA NPs exhibited excellent antioxidative properties. Next, we aimed to investigate the therapeutic effect of PDA NPs on inflammatory depression through intraperitoneal injection to the lipopolysaccharide-induced inflammatory depression model in mice. PDA NPs significantly reversed the depression-like behavior. PDA NPs was also found to reduce the peripheral and central inflammation induced by LPS, showing that alleviated splenomegaly, reduced serum inflammatory cytokines, inhibited microglial activation and restored synaptic loss. Various experiments also showed that PDA NPs had good biocompatibility both in vivo and in vitro. Our work suggested that PDA NPs may be biocompatible nano-drugs in treating inflammatory depression but their clinical application requires further study.

**Graphical Abstract:**

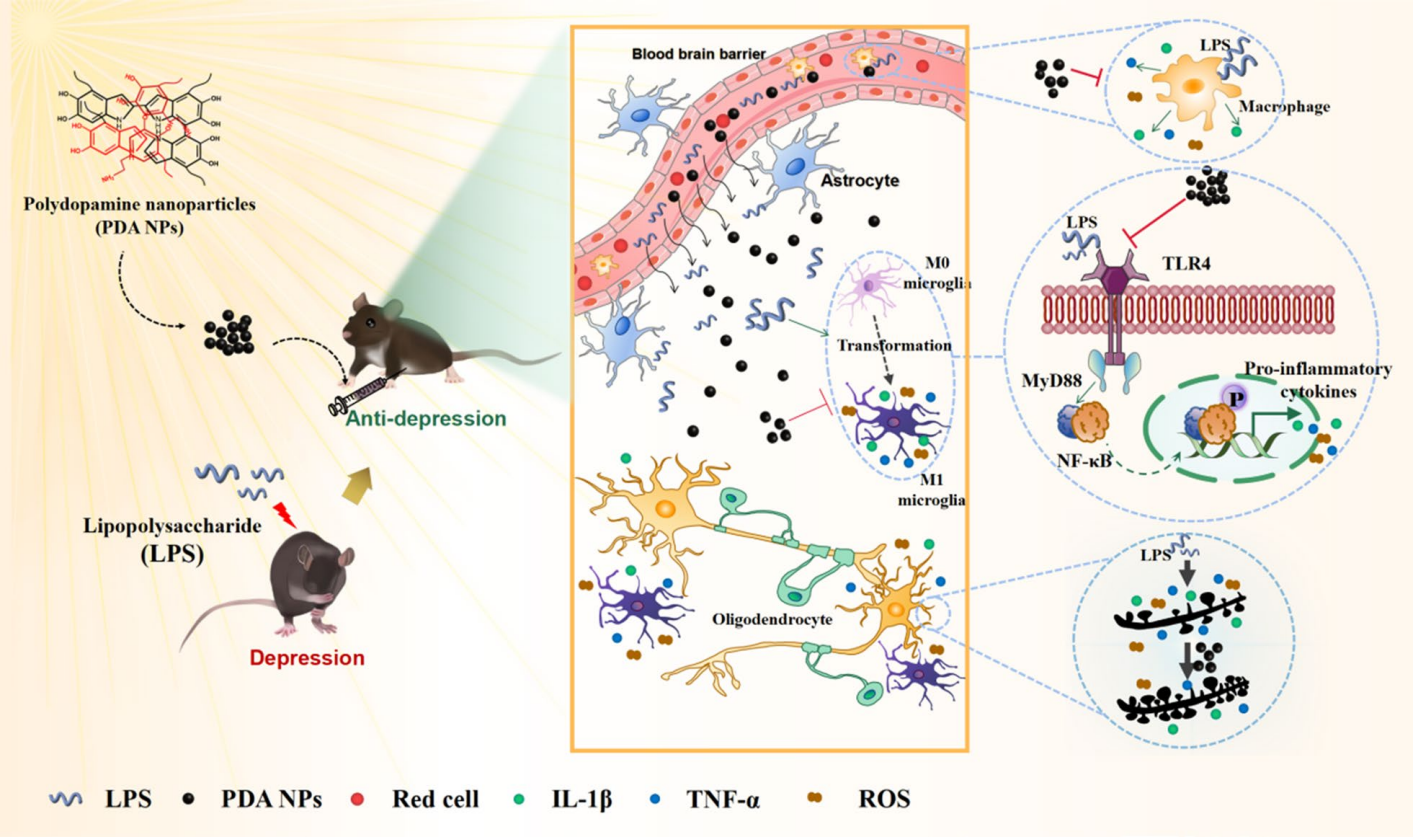

**Supplementary Information:**

The online version contains supplementary material available at 10.1186/s12951-023-01807-4.

## Introduction

Depression is a negative emotion, accompanied by thoughts of failure, hopelessness and even suicide, which is one of the most disabling psychiatric disorders, and has afflicted more than 300 million patients worldwide, especially in the course of the COVID-19 pandemic [1–3]. During the past few decades, numerous studies have shown that depression is a multifactorial disease, including but not limited to monoamines, inflammation, neurotrophies and neurogenesis, as well as (epi)genetics [4]. Among these, inflammatory depression, an emerging subtype of depression, has recently received extensive attention owing to the solid evidence of “inflammation hypothesis of depression” [5]. Previous studies have revealed that inflammatory depression is closely related to the activation of the immune system in the peripheral and central nervous system (CNS), especially central neuroinflammation [6], which is mainly mediated by the activation of microglia, the resident immune cell of the brain [7, 8]. It is well-documented that abnormal activated microglial cells trigger excessive production of inflammatory cytokines [9–11], then lead to synaptic degeneration, and ultimately result in depression-like behaviors [12, 13]. Therefore, inhibiting microglial activation and synaptic dysfunction caused by neuroinflammation may be a promising therapeutic strategy for inflammatory depression. However, the current traditional antidepressants are mainly used to interfere with abnormal neurotransmitters, such as selective serotonin reuptake inhibitors (SSRIs) and serotonin-noradrenaline reuptake inhibitors (SNRIs), and lack therapeutic effect on inflammatory depression [14]. It has been reported that abnormally increased inflammatory factors may be predictors of the treatment-resistant depression (TRD) [15]. Although several clinical trials have used anti-inflammatory drugs for antidepressant therapy, including nonsteroidal anti-inflammatory drugs (NSAIDs) and cytokine inhibitors [16–18], the therapeutic effect is still unsatisfactory due to the low permeability of blood–brain barrier (BBB) as well as the uncontrollable drug side effects [19–21]. Therefore, there is an urgent need to develop a new drug for the treatment of inflammatory depression.

Nanoparticles have been reported with the ability to cross BBB and achieve brain drug delivery [22–24]. Furthermore, the slow release of these nano drugs effectively reduces the dosing frequency, thereby limiting adverse side effects of traditional drugs [25, 26]. Polydopamine nanoparticles (PDA NPs), a melanin analogue formed by the self-polymerization of dopamine, have been widely used in the biomedical field ascribed to their high biocompatibility and biodegradability, as well as many unique chemical properties [27–29]. For example, owing to their abundant phenolic hydroxyl groups, PDA NPs demonstrated their ability to scavenge oxygen free radicals, treating periodontitis and acute pneumonia, and alleviating reactive oxygen species (ROS)-induced injury in ischemic stroke [30–32]. Collectively, PDA NPs may have a good therapeutic effect on the inflammatory depression because of their antagonism to the inflammation and ability to cross the BBB.

In the present study, we investigated the biocompatibility and antioxidative effects of PDA NPs. Additionally, PDA NPs were employed to treat inflammatory depression induced by lipopolysaccharide (LPS) and the underlying mechanisms were assessed. We aimed to develop a novel therapeutic drug for inflammatory depression.

## Results

### Preparation and characterization of PDA NPs

**Fig. 1:**
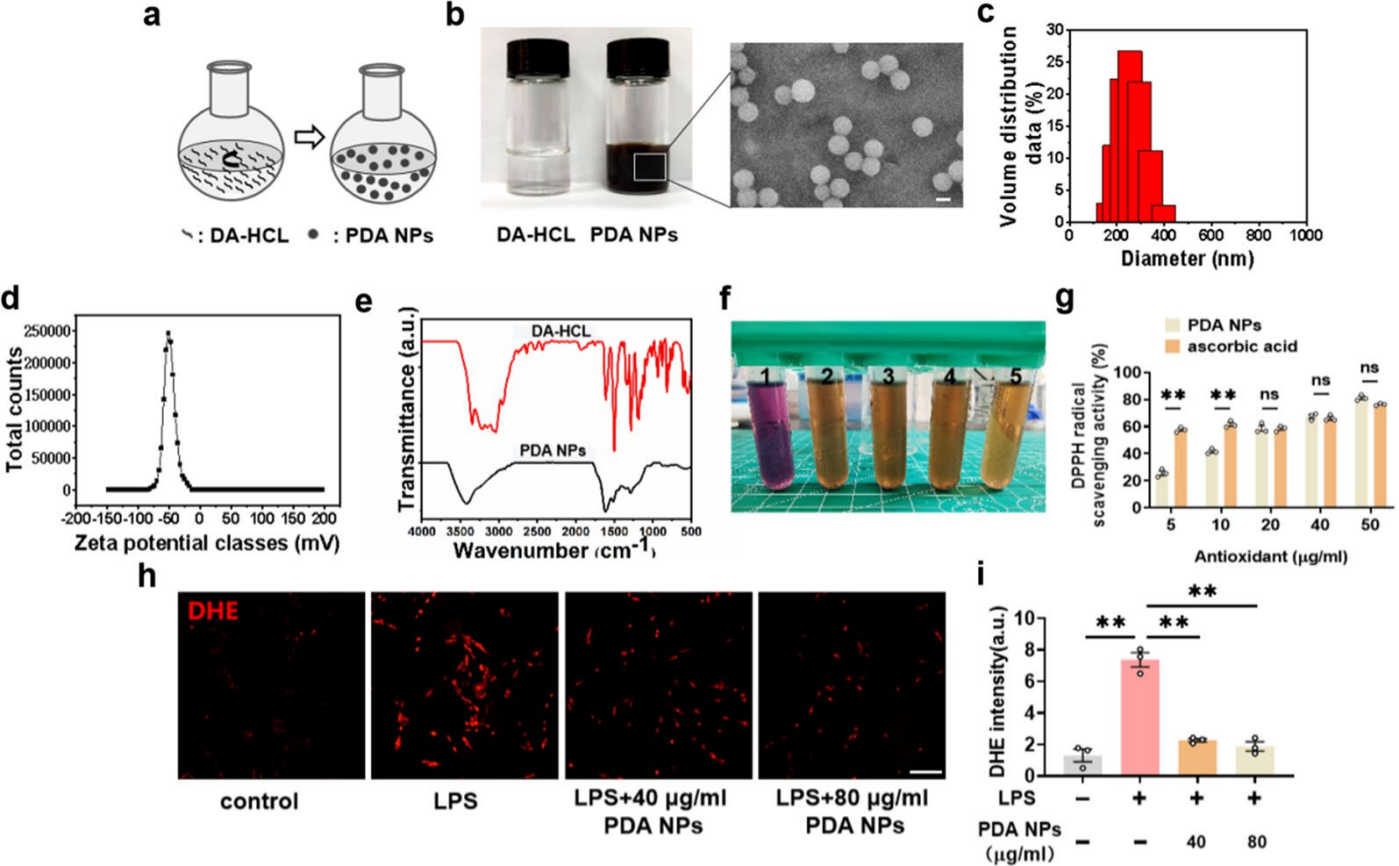
Preparation and characterization of PDA NPs. **a** Schematic illustration for the synthesis of PDA NPs. **b** Photographic and SEM images of PDA NPs (scale bar = 200 nm). **c** DLS analysis, **d** Zeta potentials analysis, and **e** FT-IR spectra of PDA NPs. **f** Photographs showing the quenching of purple-colored DPPH radicals by PDA NPs. 1: DPPH solution (0.1 mM). 2–5: the mixture DPPH solution and PDA NPs at different concentrations (10, 20, 40 and 50 μg/ml). **g** Bar graphs with dots showing the DPPH radical scavenging activity of PDA NPs and ascorbic acid [F (9, 20) = 136.8, *P* < 0.0001]. **h** Representative immunofluorescence images (scale bar = 200 μm) and **i** the corresponding bar graphs with dots showing ROS levels in the LPS-treated cells in the absence or presence of PDA NPs using DHE as the ROS probe [F (3, 8) = 68.30, *P* < 0.0001]. Data are presented as the means ± SEM. Results were analyzed by one-way ANOVA followed by Bonferroni test for post hoc comparisons. *P* < 0.05 was considered to be statistically significant. (*): *P* < 0.05, (**): *P* < 0.01 versus indicated groups; (n.s.): not significant. *DA-HCL* dopamine hydrochloride, *PDA NPs* polydopamine nanoparticles, *SEM* scanning electron microscope, *DLS* dynamic light scattering, *FT-IR* Fourier Transform Infrared, *DPPH* 2,2-Diphenyl-1-picrylhydrazyl, *DHE* dihydroethidium, *LPS* lipopolysaccharide

PDA NPs were prepared by the oxidative polymerization of dopamine hydrochloride (DA-HCL) illustrated in Fig. 1a [33]. The colorless dopamine solution turned black and brown as the reaction progressed. Typical scanning electron microscope (SEM) image revealed the monodisperse spherical structure of the resultant PDA NPs (Fig. 1b). Dynamic light scattering (DLS) results indicated that the diameter of PDA NPs was ~ 250 nm with a polydispersity index (PDI) of 0.085 (Fig. 1c). The zeta potential of PDA NPs was ~ 49 mV (Fig. 1d). To further confirm the successful formation of PDA NPs, Fourier Transform Infrared Spectroscopy (FTIR) for the PDA NPs and DA-HCL was conducted. As shown in Fig. 1e, the indole-related structure located around 1600 cm^−1^ was formed in PDA NPs but nonexistent in DA-HCL. Because of the decrease of aromatic hydrogen and aromatic nucleus, the absorption band between 800 cm^−1^ and 700 cm^−1^ became weaker in PDA NPs than that of DA-HCL. These results indicated the successful formation of PDA NPs.

To verify the ability to scavenge oxygen free radicals of PDA NPs, we conducted the antioxidant capacity test in vitro by 2, 2-diphenyl-1-pyridinium hydrazine (DPPH) assay [34, 35]. The scavenging oxygen free radicals could be obviously responded by the apparent color loss of DPPH solution (Fig. 1f). The quantitative results indicated that there was no difference between PDA NPs and ascorbic acid, a classical free radical scavenging material [33], in the scavenging ability of free radicals when the concentration increased to 20 μg/ml (Fig. 1g). Next, to further evaluate the ability of PDA NPs to scavenge ROS in cells, we used dihydroethidium (DHE) as a probe, which can be taken up into the cells, producing ethidium with red fluorescent under the oxidation of intracellular ROS [36, 37]. In our experiment, as revealed by fluorescence images of DHE stained cells, the pretreatment of PDA NPs could efficiently remove the drastic increase of ROS level in PC12 cells caused by LPS exposure (Fig. 1h, i). Therefore, PDA NPs showed significant antioxidant activity to suppress the production of toxic ROS. Altogether, the above results suggested that PDA NPs exhibited excellent antioxidation.

### PDA NPs improved anxiety- and depression-like behaviors

**Fig. 2:**
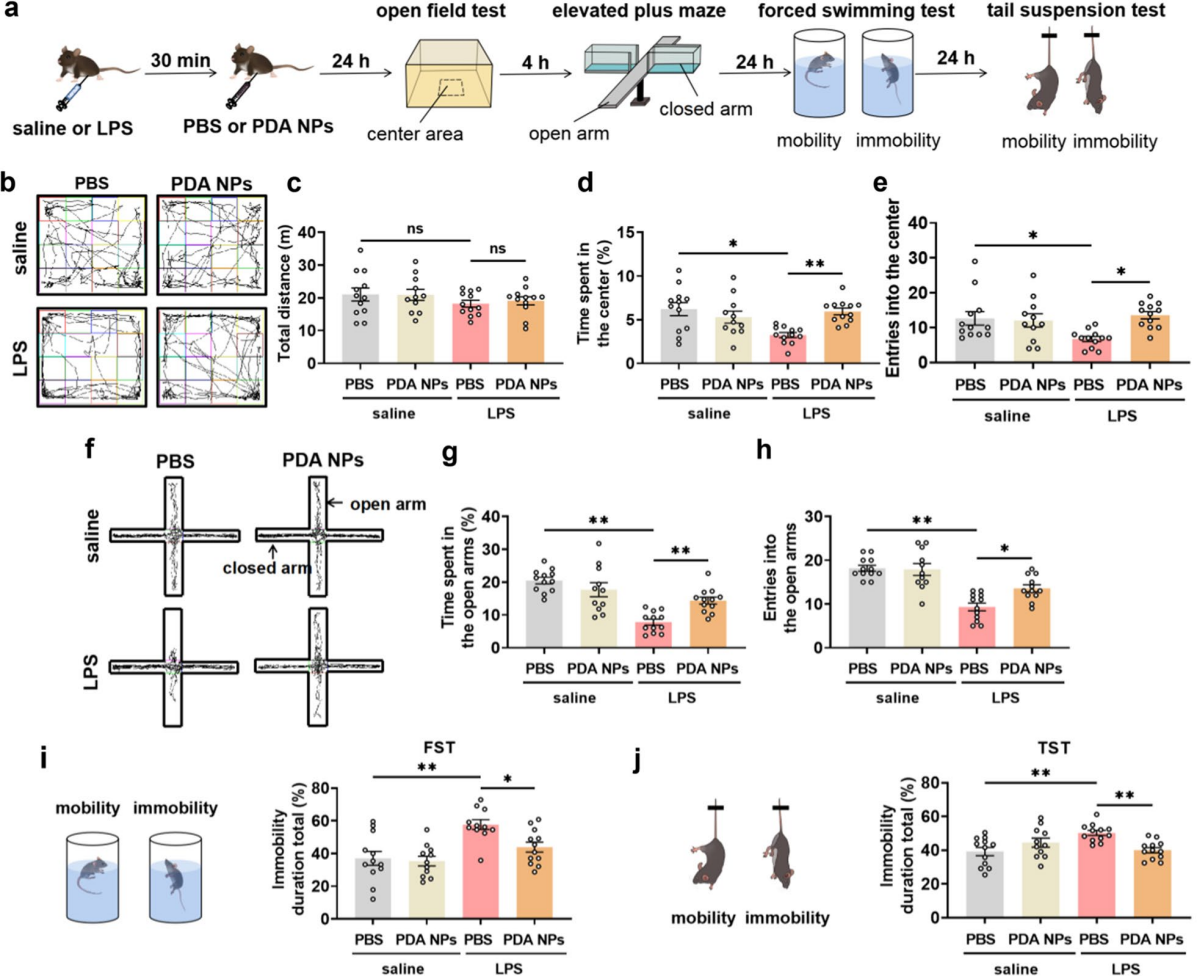
PDA NPs improved anxiety- and depression-like behaviors. **a** Schematic illustration of the experimental procedure. LPS was intraperitoneal injected into mice to induce inflammatory depression, which was improved by PDA NPs. The therapeutic effects can be evaluated by anxiety and depression-like behavior tests, including open field test (OFT), elevated plus maze (EPM), forced swimming test (FST) and tail suspension test (TST). **b** Representative activity tracking and the corresponding bar graphs with dots showing **c** total distances as well as percentage of **d** time spent in and **e** entries into the center during the 5 min in the OFT [Distances: F (3, 43) = 0.8294, *P* = 0.4850; Time: F (3, 43) = 6.064, *P* = 0.0015; Entries: F (3, 43) = 4.328, *P* = 0.0094]. **f** Representative activity tracking and the corresponding bar graphs with dots showing percentage of **g** time spent in and **h** entries into the open arms of the maze [Time: F (3, 43) = 17.00, *P* < 0.0001; Entries: F (3, 43) = 19.51, *P* < 0.0001]. **i** Bar graphs with dots showing percentage of immobility time in FST and **j** TST [FST: F (3, 42) = 8.686, *P* = 0.0001; TST: F (3, 43) = 5.977, *P* = 0.0017]. Data are presented as the means ± SEM (n = 11–12 in each group). Results were analyzed by one-way ANOVA followed by Bonferroni test for post hoc comparisons. (*): *P* < 0.05, (**): *P* < 0.01 versus indicated groups; (n.s.): not significant. *LPS* lipopolysaccharide, *PBS* phosphate-buffered saline, *PDA NPs* polydopamine nanoparticles, *FST* forced swimming test, *TST* tail suspension test

Encouraged by the effective anti-oxidation ability of PDA nanoparticles in vitro, we then studied their potential usages in the inflammation-related depression which was induced by intraperitoneal (i.p.) injection of LPS (0.83 mg/kg). PDA NPs (10 mg/kg) were i.p. injected at 30 min after LPS injection (Fig. 2a), which was a better treatment plan through our early exploration (Additional file 1: Figure S1).

Depressed patients are always accompanied by anxiety-like behaviors [38], so the rescue of depression and anxiety-like behaviors by PDA NPs was jointly considered in our behavioral experiments. In order to explore the effects of PDA NPs on anxiety-like and depression-like behaviors caused by LPS injection, we conducted open field test (OFT), elevated plus maze (EPM), forced swimming test (FST) and tail suspension test (TST), as showed in Fig. 2a. The OFT demonstrated that there was no statistical difference in the total distance among all groups, which ruled out the possibility that the behavioral results were due to locomotor function (Fig. 2b, c). Noticeably, significant decreases were seen in the ratio of time spent in the center and open arms and the entries into the center and open arms in the LPS + PBS group compared with the saline + PBS group, but the decreases were obviously reversed in the LPS + PDA NPs group (Fig. 2d–h). We further performed forced swimming test (FST) and tail suspension test (TST), which are used to measure the depression-like behavior [13, 39]. Compared with the saline + PBS group, the immobility time of the LPS + PBS group in both FST and TST increased significantly, but the increased time was obviously decreased in the LPS + PDA NPs group (Fig. 2i, j). These behavior results demonstrated that PDA NPs could effectively rescue the anxiety- and depression-like behaviors induced by LPS. Together, the above results suggested a potential application of PDA NPs in patients with or at risk of inflammation-related depression.

### PDA NPs reversed peripheral inflammation

**Fig. 3:**
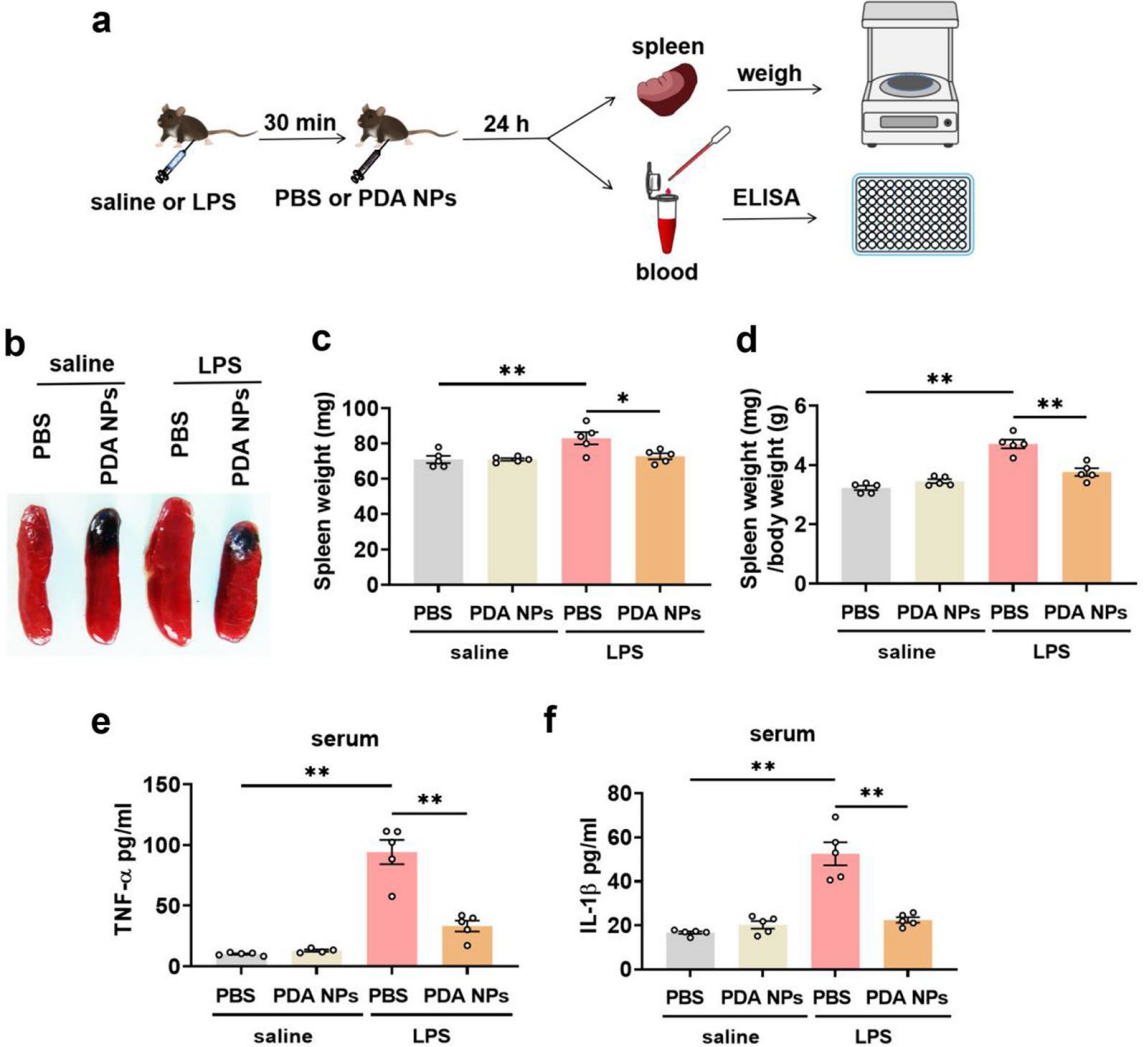
PDA NPs reversed peripheral inflammation. **a** Schematic diagram showing the measurement of spleen weight and the detection of inflammatory factors in serum by ELISA assay. **b** Representative picture of spleen and the corresponding bar graphs with dots showing **c** spleen weight [F (3, 16) = 6.798, *P* = 0.0036] and **d** the ratio of spleen weight/body weight [F (3, 16) = 35.10, *P* < 0.0001] as well as the serum levels of **e** TNF-α [F (3, 15) = 45.95, P < 0.0001] and **f** IL-1β [F (3, 16) = 33.20, *P* < 0.0001]. Data are presented as the means ± SEM (n = 4–5/group). Results were analyzed by one-way ANOVA followed by Bonferroni test for post hoc comparisons. (*): *P* < 0.05, (**): *P* < 0.01 versus indicated groups; (n.s.): not significant. *LPS* lipopolysaccharide, *PBS* phosphate-buffered saline, *PDA NPs* polydopamine nanoparticles, *ELISA* enzyme-linked immune sorbent assay, *TNF-α* tumor necrosis factor alpha, *IL1-β* interleukin-1 beta

The behavioral abnormalities induced by LPS exposure are due to the activation of the immune system in the peripheral and CNS [6, 40]. Therefore, we first investigated whether PDA NPs can suppress the peripheral inflammatory response (Fig. 3a). It has been reported that LPS-induced systemic inflammation was always accompanied by the increased weight of the spleen, an important immune organ [41]. In line with previous studies, we found that the weight of spleen collected 24 h after LPS injection was dramatically increased. As expected, administration of PDA NPs remarkably reversed LPS-induced splenomegaly and decreased the ratio of spleen weight to body weight (Fig. 3b–d). In addition, to further confirm the peripheral anti-inflammatory effects of PDA NPs, blood samples were also collected 24 h after LPS injection for the measurement of inflammatory cytokines including TNF-α and IL-1β. The results indicated that PDA NPs could significantly alleviate the increased serum levels of TNF-α and IL-1β caused by LPS exposure (Fig. 3e, f). Therefore, both spleen weight and serum cytokine data revealed that the peripheral inflammatory response of inflammatory depression was greatly attenuated by treatment with PDA NPs.

### PDA NPs restrained the pro-inflammatory transformation of microglia via the TLR4/NF-κB signaling pathway

**Fig. 4:**
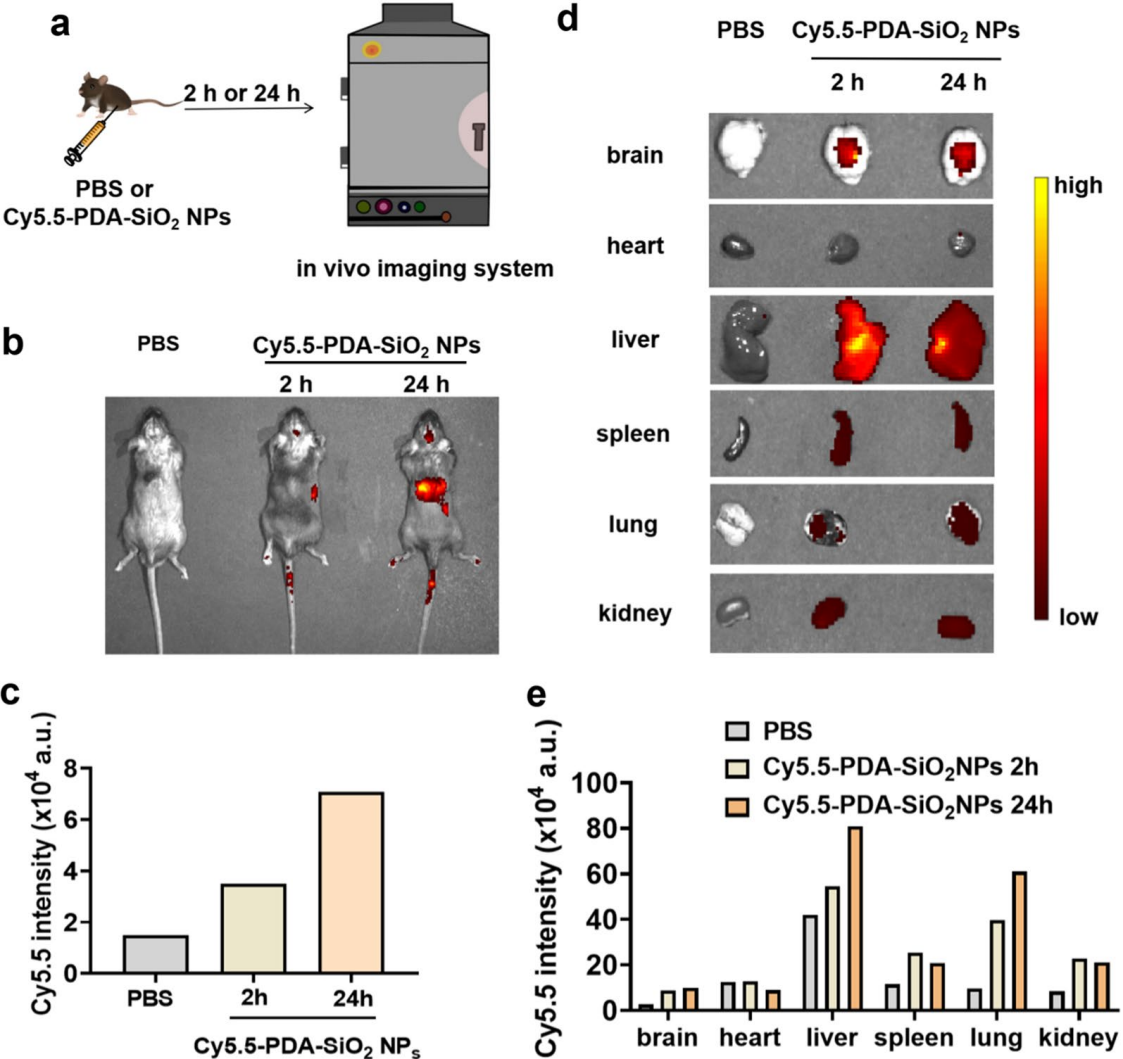
PDA NPs successfully penetrated BBB to enter the brain. **a** Schematic illustration showing the experimental procedures for fluorescent images of mice. **b** Representative Cy5.5 fluorescence imaging showing the whole-body distribution of PDA NPs in mice after 2 h and 24 h of Cy5.5-PDA-SiO_2_ NPs injection. **c** Quantification of Cy5.5 intensity in brain 2 h and 24 h after injection. **d** The Cy5.5 fluorescent images and **e** the corresponding bar graphs showing the organ distribution of PDA NPs. *PBS* phosphate-buffered saline, *Cy5.5-PDA-SiO*_*2*_* NPs* cyanine 5.5-polydopamine-silica nanoparticles

Secondary neuroinflammation of the brain caused by peripheral inflammation is the main cause of inflammatory depression [6, 40, 42]. Therefore, it is necessary to explore the central role of PDA NPs. Before the investigation of the central effects of PDA NPs, we first applied in vivo imaging to confirm the ability of PDA NPs cross the BBB (Fig. 4a). We used Cy5.5-PDA-SiO_2_ NPs to imitate the labeled PDA NPs in vivo (Additional file 1: Figure S2a). The results of transmission electron microscopy (TEM) and DLS analysis showed that Cy5.5-PDA-SiO_2_ NPs were spherical particles with a mean size of 244 nm and a PDI of 0.119 (Additional file 1: Figure S2b, c) as well as the Zeta potentials were − 48 mV (Additional file 1: Figure S2d), which were similar to PDA NPs (Fig. 1c, d). Therefore, it is feasible to label the distribution of PDA NPs in vivo with Cy5.5-PDA-SiO_2_ NPs. As expected, when the Cy5.5-PDA-SiO_2_ NPs were intraperitoneally injected, especially after 24 h, an obvious Cy5.5 fluorescence signal could be detected in the brain, indicating that Cy5.5-PDA-SiO_2_ NPs could efficiently cross the BBB (Fig. 4b, c). By imaging the organs after sacrificing the mice, the Cy5.5-PDA-SiO_2_ NPs were further confirmed to be able to enter the brain. Moreover, obvious fluorescent signals appeared in the liver, kidney and spleen (Fig. 4d, e), which indicated that Cy5.5-PDA-SiO_2_ NPs might be partly metabolized by the liver and kidney, while the distribution of the spleen gave solid evidence that PDA NPs have a certain effect on the spleen, consistent with our previous study (Fig. 3b–d).

**Fig. 5:**
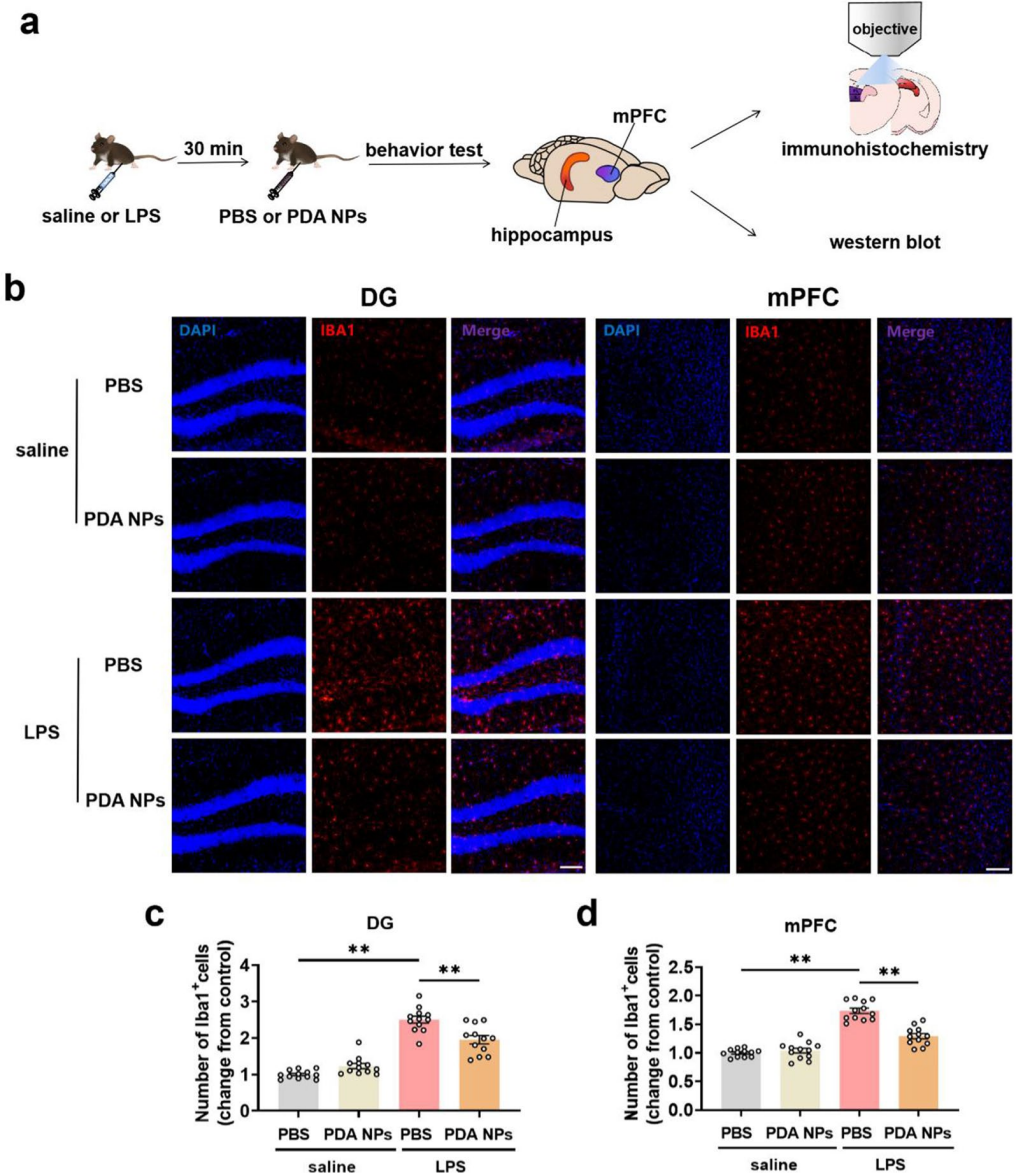
PDA NPs reversed the increased microglial numbers induced by LPS in the hippocampus and mPFC. **a** Schematic diagram showing hippocampal and mPFC tissue analysis by using immunohistochemistry or western blot after behavior test. **b** Representative immunofluorescence images and the corresponding bar graphs with dots showing Iba1^+^ microglia (red) in the **c** DG of hippocampus [F (3, 44) = 66.79,* P* < 0.0001] as well as **d** mPFC [F (3, 44) = 70.28, P < 0.0001] (12 images from 3 mice in each group). Scale bar = 100 μm. Data are presented as the means ± SEM. Results were analyzed by one-way ANOVA followed by Bonferroni test for post hoc comparisons. (*): *P* < 0.05, (**): *P* < 0.01 versus indicated groups. *LPS* lipopolysaccharide, *PBS* phosphate-buffered saline, *PDA NPs* polydopamine nanoparticles, *mPFC* medial prefrontal cortex, *DG* dentate gyrus of hippocampus, *IBA1* ionized calcium binding adaptor molecule 1

**Fig. 6:**
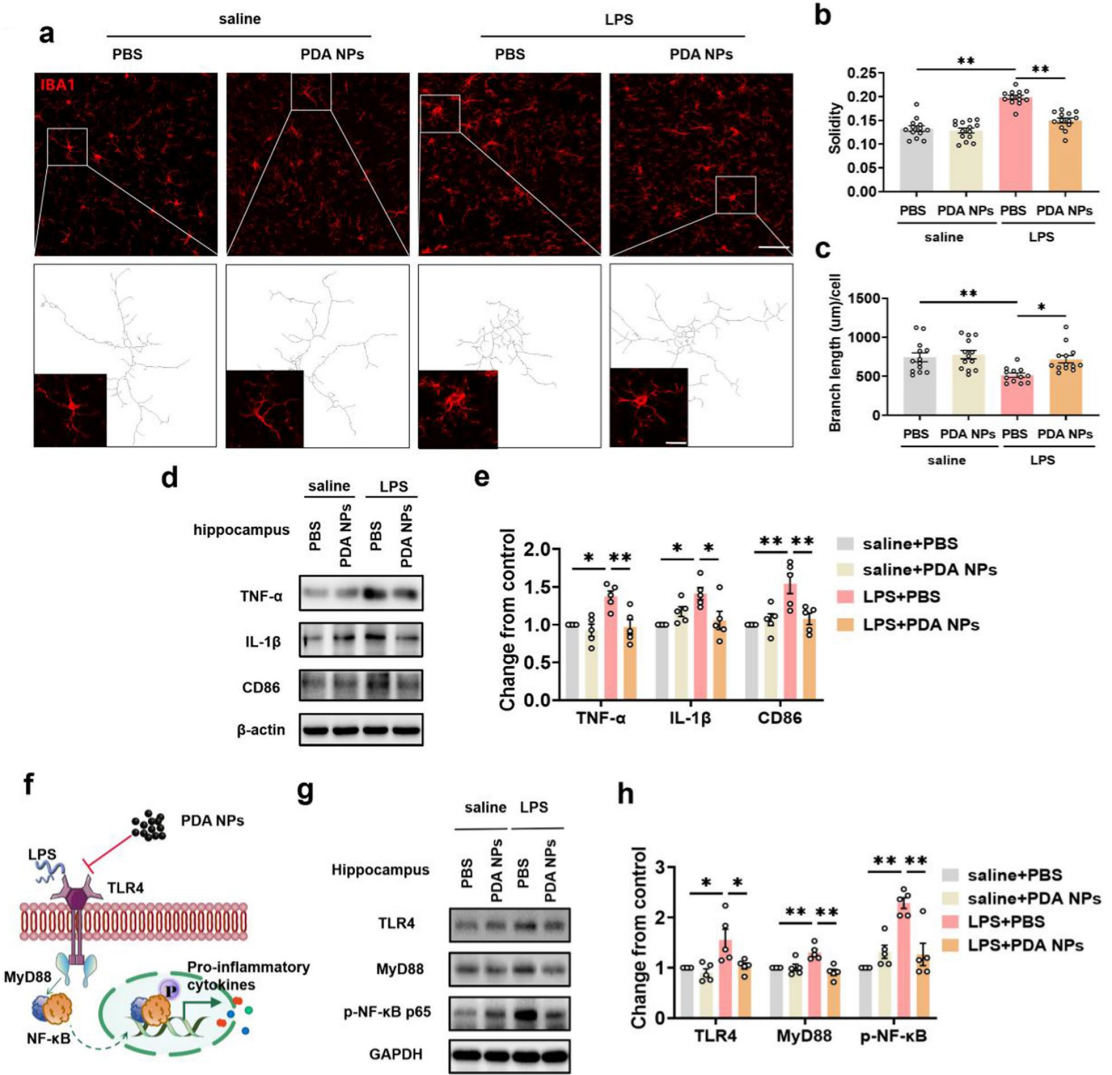
PDA NPs restrained the pro-inflammatory transformation of microglia via the TLR4/NF-κB signaling pathway. **a** Confocal fluorescence images showing the morphology of microglia. Inset of a: fluorescence image of single microglia under zoom and skeletonization. Scale bar = 50 μm (overview) and 5 μm (inset). Quantification of Iba1 ^+^ cell **b** solidity [F (3, 49) = 39.23, *P* < 0.0001] and **c** branch length [F (3, 47) = 5.904, *P* = 0.0017] (12 images from 3 mice in each group). **d** Representative western blot and **e** the corresponding bar graphs with dots showing TNF-α, IL-1β and CD86 expression levels in the hippocampus [TNF-α: F (3, 16) = 8.289, *P* = 0.0015; IL-1β: F (3, 16) = 5.384, *P* = 0.0094; CD86: F (3, 16) = 8.607, *P* = 0.0012] (n = 5/group). **f** The initial mechanism of PDA NPs restraining LPS induced microglial activation. **g** Representative western blot and **h** the corresponding bar graphs with dots showing TLR4, MyD88 and p-NF-κB p65 expression levels in the hippocampus [TLR4: F (3, 16) = 6.536, *P* = 0.0043; MyD88: F (3, 16) = 9.799, *P* = 0.0007; p-NF-κB p65: F (3, 16) = 16.35, *P* < 0.0001[ (n = 5/group). Data are presented as the means ± SEM. Results were analyzed by one-way ANOVA followed by Bonferroni test for post hoc comparisons. (*): *P* < 0.05, (**): *P* < 0.01 versus indicated groups. *LPS* lipopolysaccharide, *PBS* phosphate-buffered saline, *PDA NPs* polydopamine nanoparticles, *TNF-α* tumor necrosis factor alpha, *IL1-β* interleukin-1 beta, *CD86* cluster of differentiation 86, *TLR4* toll Like receptor 4, *MyD88* myeloid differentiation factor 88, *p-NF-κB* phospho-nuclear factor kappa-B

Having successfully demonstrating the BBB permeability of PDA NPs, we then estimated their central effects. It has been reported that the abnormal activation of microglia caused by acute inflammatory stimulation can result in depression [42, 43]. To this end, we conducted immunohistochemistry using an antibody of Iba1, a recognized maker for microglia cells, indicating that PDA NPs could reverse the significantly increased microglial numbers induced by LPS in the hippocampus and mPFC (Fig. 5a–d and Additional file 1: Figure S3), which are two critical brain regions responsible for emotion regulation [44]. Subsequently, we employed skeletonization analysis to observe the morphology of microglia. Consistent with previous studies [45, 46], we found that microglia in the LPS + PBS group had significantly larger cell bodies and shorter branches than the normal saline group (Fig. 6a, c). In addition, we also conducted solidity analysis, that is, the ratio of cell body area to branch area can reflect the microglial phagocytic function [47]. The significantly increased solidity in LPS + PBS group verified that LPS exposure led to microglial activation. As expected, PDA NPs could significantly reverse the abnormal morphological changes of these microglia induced by LPS (Fig. 6b, c). The results of the number and morphology analysis of microglia showed that PDA NPs could effectively reverse the activation of microglia in the CNS induced by LPS.

As we know, activated microglia display two main phenotypes, namely, the pro-inflammatory M1 phenotype and the anti-inflammatory M2 phenotype. Among them, M1 microglia secrete excessive inflammatory factors and ROS, leading to the death of normal cells, thereby causing damage to the body [48, 49]. As a result, we further used western blot analysis to verify that PDA NPs could mitigate significantly increased TNF-α, IL-1β and CD86 in the hippocampus and medial prefrontal cortex induced by LPS, which are specific markers of M1 microglia (Fig. 6d, e and Additional file 1: Figure S4).

M1 microglia break the antioxidant/oxidant balance to promote the production of ROS, which consequently led to oxidative stress [43, 50]. Therefore, we also demonstrated that PDA NPs significantly inhibited the oxidative stress induced by M1 microglia, as indicated by significantly reducing the MDA content in the brain (Additional file 1: Figure S5a), which is a product of lipid peroxidation, reflecting the level of oxidative stress [51]. Antioxidant enzyme SOD represents the balance of pro-oxidants and antioxidants in the process of oxidative stress [52]. Although PDA NPs could not reverse the activity of SOD, it showed a strong increasing tendency towards statistical significance (Additional file 1: Figure S5b). In addition, to further confirm the protective role of PDA NPs, we investigated DNA damage caused by oxidative stress. 8-hydroxydeoxyguanosine (8-OHdG) is the typical marker that indicates DNA damage [53]. The results of immunohistochemistry revealed that LPS-treated mice presented an upregulation of 8-OHdG in the hippocampus and mPFC, whereas PDA NPs administration reversed the upregulation and alleviated the effect of LPS (Additional file 1: Figure S5c-h). Overall, the above results revealed that PDA NPs could effectively inhibit the activation of pro-inflammatory M1 microglia induced by LPS to alleviate the excessive production of inflammatory factors and reactive oxygen species, which might be the reason that PDA NPs could prevent LPS induced depression.

We next examined the initial mechanism of PDA NPs restraining LPS induced microglial activation. It is generally believed that LPS can activate microglia by binding to toll-like receptor 4 (TLR4) expressed on their surface, and then the adaptor protein MyD88 (myeloid differentiation primary response gene 88) of TLR4 activates its downstream NF-κB signal pathway, which then mediates the production of pro-inflammatory cytokines and ROS (Fig. 6f) [54].

As reported, the results of western blotting showed that the expression of TLR4, MyD88 and p-NF-κB p65 significantly increased compared with saline treatment in hippocampus and mPFC after LPS exposure. As expected, the administration of PDA NPs remarkably reduced these protein levels, suggesting largely inhibited the activation of microglia through TLR4/NF-κB signaling pathway (Fig. 6g, h and Additional file 1: Figure S6). However, the detailed underlying mechanism that PDA NPs act on TLR4/NF-κB signaling pathway remains unclear, which needs further investigation.

Additionally, in order to eliminate the accompanying changes caused by the nanometer effect of PDA NPs, we introduced silica nanoparticles, of which particle size are similar to PDA NPs (Additional file 1: Figure S7). As shown in Additional file 1: Figure S8, the silica nanoparticles could not reverse the activation of TLR4-MyD88 induced by LPS, revealing that the antidepressant effect of PDA NPs was indeed dependent on their own anti-inflammation and antioxidant properties, unrelated to the accompanying phenomena of nanoparticles.

Collectively, the above results suggested that PDA NPs suppressed the pro-inflammatory transformation of microglia after LPS injection via the TLR4/NF-κB signaling pathway.

### PDA NPs alleviated the impairment of synaptic structures

**Fig. 7:**
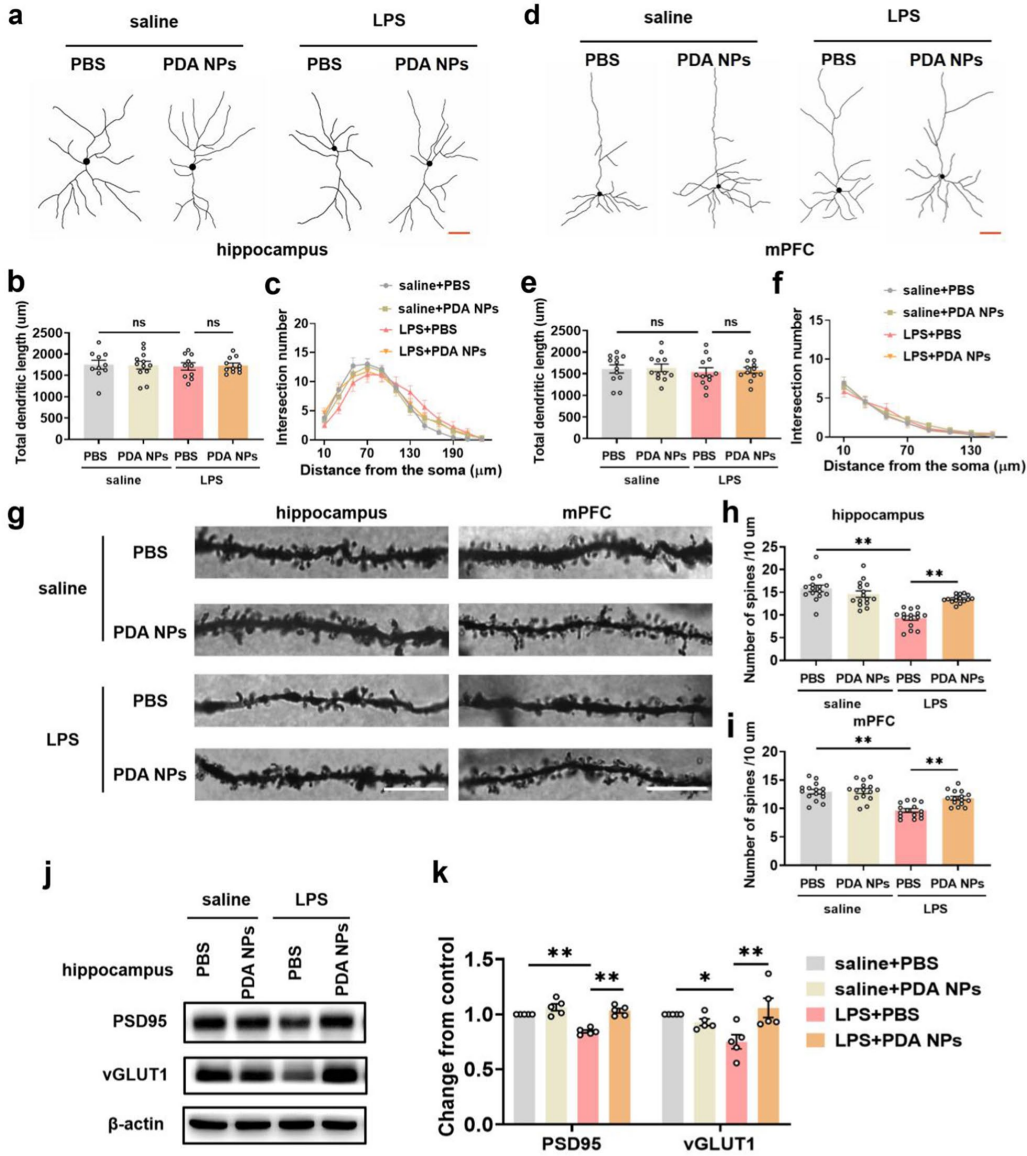
PDA NPs alleviated the impairment of synaptic structures. **a** Representative camera tracings and the corresponding bar graphs with dots showing **b** total dendritic length [F (3, 39) = 0.04098, *P* = 0.9888] and **c** intersection numbers of hippocampal pyramidal neurons by Golgi-Cox staining (10–12 neurons from 3 mice per group). **d** Representative camera tracings and the corresponding bar graphs showing **e** total dendritic length [F (3, 44) = 0.2182, *P* = 0.8832] and **f** intersection numbers of pyramidal neurons in the mPFC (12 neurons from 3 mice per group). Scale bar = 50 μm (A, D). **g** Representative images and the corresponding quantification showing neuronal dendrites spine numbers per 10 mm in the **h** hippocampus [F (3, 56) = 24.79, *P* < 0.0001] and **i** mPFC [F (3, 56) = 17.99, *P* < 0.0001] (15 dendrites from 3 mice per group). Scale bar = 10 μm. **j** Representative western blot and **k** the corresponding bar graphs with dots showing PSD-95 [F (3, 16) = 23.84, *P* < 0.0001] and vGLUT1 [F (3, 16) = 5.498, *P* = 0.0086] expression levels in the hippocampus (n = 5–6/group). Data are presented as the means ± SEM. Results were analyzed by one-way ANOVA followed by Bonferroni test for post hoc comparisons. (*): *P* < 0.05, (**): *P* < 0.01 versus indicated groups; (n.s.): not significant. *LPS* lipopolysaccharide, *PBS* phosphate-buffered saline, *PDA NPs* polydopamine nanoparticles, *mPFC* medial prefrontal cortex, *PSD95* postsynaptic density protein 95, *vGLUT1* vesicular glutamate transporter 1

Excessive neuroinflammation caused by abnormal activation of microglia leads to synaptic degeneration, which in turn reduces neuronal connections and eventually leads to depression like behavior [12, 49]. Therefore, we conducted Golgi staining to observe the effect of PDA NPs on neurons according to the method previously described. The results were shown in Fig. 7a–f and demonstrated that there was no difference in the total dendritic length and intersection numbers of neurons in the hippocampus and mPFC among the four groups. But surprisingly, PDA NPs significantly inhibited the loss of hippocampal dendritic spines induced by LPS, and did not affect the density of spines in saline injected mice, as shown in Fig. 7g–i. The same changes were also observed in the mPFC brain region.

Meanwhile, western blot was further conducted to verify that in the two brain regions, administration of PDA NPs markedly attenuated the reduced levels of presynaptic vGLUT1 and postsynaptic PSD-95 induced by LPS (Fig. 7j, k and Additional file 1: Figure S9). Synaptic function depends on the integrity of its structure, while previous studies have shown that LPS-induced depressive-like behaviors are closely related to synaptic loss and dysfunction [13, 55]. Based on the above findings, the antidepressant effect of PDA NPs might be related that it alleviated the impairment of synaptic structures induced by LPS.

### Biocompatibility of PDA NPs

**Fig. 8:**
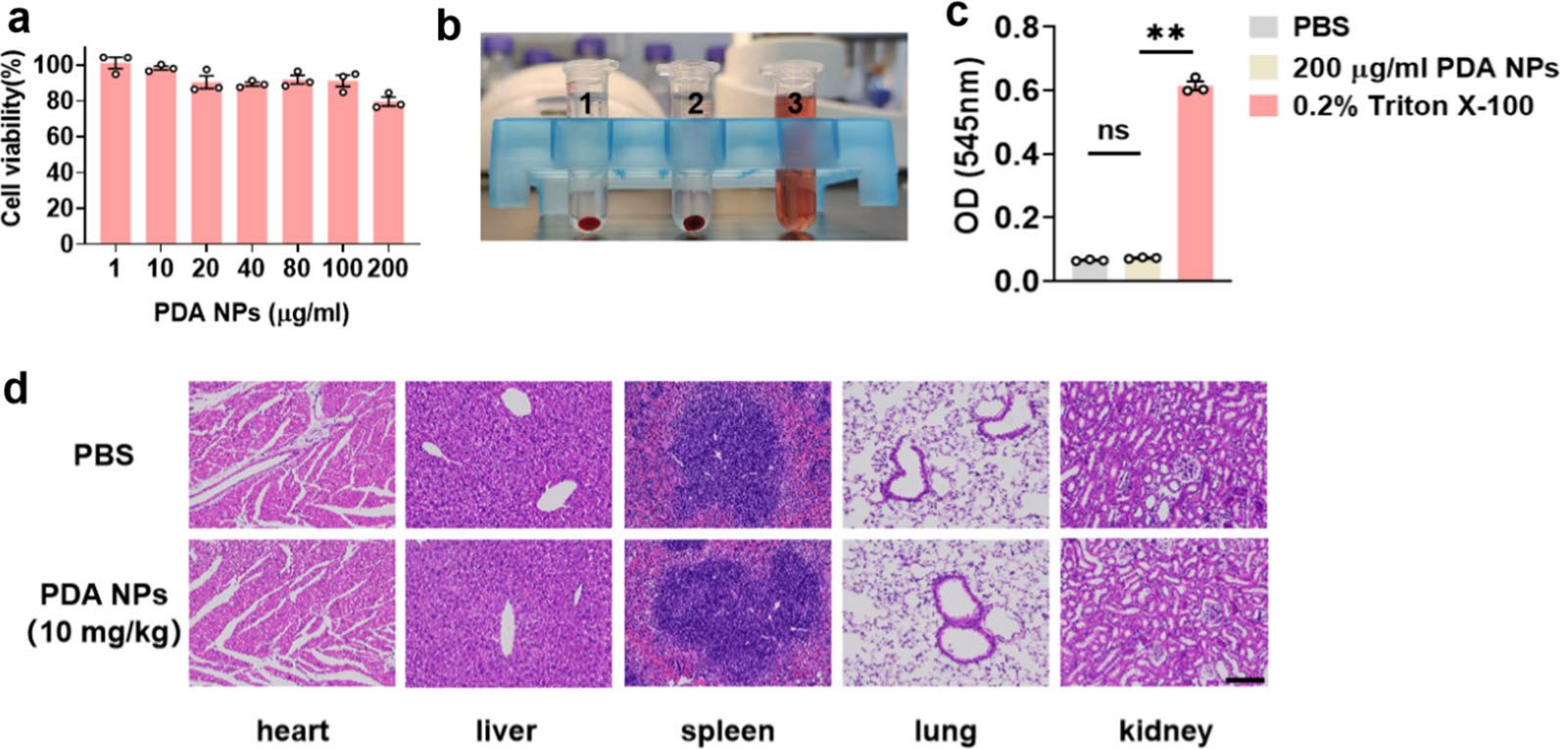
PDA NPs exhibited excellent biocompatibility both in vitro and in vivo. **a** Bar graphs with dots showing viability of PC12 cells incubated with PDA NPs for 24 h [F (5, 12) = 2.336, *P* = 0.1061]. **b** Representative picture showing hemolytic reaction of red blood cells in different solutions: 1. PBS; 2. 200 μg/ml PDA NPs; 3. 0.2% Triton X-100. **c** Bar graphs with dots showing blood compatibility of PDA NPs [F (2, 6) = 1553, *P* < 0.0001]. **d** Representative histological images of main organs post treatments. Scale bar = 100 μm. Data are presented as the means ± SEM (n = 3 per group). Results were analyzed by one-way ANOVA followed by Bonferroni test for post hoc comparisons. (**): *P* < 0.01 versus indicated groups; (n.s.): not significant. *PBS* phosphate-buffered saline, *PDA NPs* polydopamine nanoparticles

Prior to the use of PDA NPs in vivo for depression, it was important to investigate their biocompatibility [56]. Cell counting kit (CCK8) assay was carried out to detect the viability of PC-12 cells towards PDA NPs. As shown in Fig. 8a, PC12 cells showed good cell viability after 24 h of incubation with PDA NPs (1, 10, 20, 40, 80 and 100 μg/ml). Notably, the cellular safety of PDA NPs was concentration dependent. High concentrations of PDA NPs were found to have an effect on cell proliferation (Additional file 1: Figure S10). In addition, hemolytic assay was considered to estimate the blood compatibility of PDA NPs. Even at a high concentration of 200 μg/ml, no obvious hemolysis occurred after 4 h co-incubation (Fig. 8b, c). More importantly, to verify the overall safety of PDA nanoparticles in vivo, HE staining was used to observe the morphology of heart, liver, spleen, lung and kidney tissues. The results revealed that PDA NPs (10 mg/kg) did not cause significant damage to the vital organs (Fig. 8d). As previously mentioned, PDA NPs exhibited good biocompatibility both in vivo and in vitro.

## Discussion

In this study, melanin-like PDA NPs were successfully prepared by the simplest one-step synthesis method. In vitro, PDA NPs exhibited excellent antioxidation. In vivo, PDA NPs significantly reversed the depression behavior of mice by suppressing LPS-induced microglial activation and synaptic loss through TLR4/NF-κB signaling. Moreover, PDA NPs showed good biocompatibility both in vivo and in vitro. Therefore, our study demonstrated that PDA NPs might be a potential excellent nano-drug in treating inflammatory depression.

Currently, treatments available for depression mainly regulate monoamine neurotransmitters in the brain, among which selective serotonin reuptake inhibitors (SSRIs) and serotonin-noradrenaline reuptake inhibitors (SNRIs) are widely used as first-line antidepressants [57]. Nevertheless, about 50% of patients still have no effective relief from their depressive symptoms due to poor bioavailability, delayed therapeutic onset, drug toxicity and dose-dependent adverse effects [58]. Recently, nanotechnology-based drug delivery strategies have been employed to improve the above limitations [59]. Traditional drugs exhibit low BBB permeability, resulting in low bioavailability [60]. Nano-drugs, however, can overcome this defect because they can penetrate endothelial cells through cellular phagocytosis and then cross the BBB [61, 62], which is essential for antidepressants. Additionally, Ligand-complexed nanoparticles can target specific receptors to improve antidepressant efficacy and limit drug toxicity [63]. Furthermore, biopolymers and nanocarriers can be designed to enhance controlled release of antidepressants, thereby reducing the frequency of administration and adverse effects [64, 65]. Integrally, the application of nano therapy in depression has gained significant attention.

PDA NPs have many unique chemical properties, such as excellent chelating capacity to metal ions, strong molecular adsorption ability and high biocompatibility, which makes them widely used in nuclear imaging, drug delivery, molecular imaging and other biomedical fields [27, 66]. Moreover, due to their abundant phenolic hydroxyl groups, PDA NPs can also act as excellent free radical scavengers to treat damage induced by ROS or acute inflammation [30, 31]. In this study, we proved that PDA NPs exhibited excellent antioxidation in vitro. Increasing evidence indicates the critical role of inflammation and oxidative stress in the development of depression [12, 67]. Hence, PDA NPs may be suitable for the treatment of inflammatory depression. In the present study, we systematically administrated PDA NPs after LPS injection, and anxiety- and depression-like behaviors were significantly improved. Moreover, we demonstrated that PDA NPs had good biosafety both in vivo and in vitro. Taken together, these data suggested that PDA NPs have clinical translational value in the treatment of patients with or at risk for inflammatory depression.

Previous studies have shown that inflammatory depression is closely related to the activation of the immune system in the peripheral and CNS [40]. It has also been reported that peripheral inflammation is closely associated with spleen weight as well as serum inflammatory factors [41]. Our study extended previous findings by showing that splenomegaly and elevated serum levels of IL-1β and TNF-α in LPS models could be rescued by PDA NPs treatment. In addition, our study confirmed that PDA NPs could cross the BBB due to their unique nanometer effect, which is critical to their antagonism to neuroinflammation. It is well known that neuroinflammation caused by peripheral inflammation is the main cause of inflammatory depression [6, 42]. Microglia are resident immune cells of the CNS and major mediators of neuroinflammation. Under normal conditions, microglia are in a resting state, that is, their cell bodies are small and highly branched. However, when stress and injury occur, the number of microglia increases on the one hand, and their morphology changes on the other hand, mainly manifested as the relative increase of cell bodies and the contraction of branches [45, 46]. Therefore, the number and morphological changes of microglia are well correlated with their activation state. Consistent with these previous studies, we also found that LPS caused overactivation of microglial cells in the brain, and these abnormal changes were rescued by PDA NPs. These data revealed that PDA NPs could effectively inhibit neuroinflammation, which may account for their antidepressant effect. Furthermore, the classical molecular mechanism mediating microglial activation is TLR4/NF-κB signaling pathway [54]. Here, we proved that TLR4 was reduced with the administration of PDA NPs, which further decreased MyD88 and phosphorylation of p65. These data suggested that PDA NPs might improve inflammatory depression by modulating TLR4/NF-κB signaling pathway, although the detailed underlying mechanisms are currently unknown.

Numerous studies have indicated that synaptic damage caused by excessive neuroinflammation exerts a critical part in the pathogenesis of depression [12, 49]. In this study, similar results were observed that synaptic structures were significantly impaired in LPS-induced depressed mice. Interestingly, this impairment could be restored by PDA NPs. Synaptic function depends on the integrity of its structure [55]. It has also been reported that excessive inflammatory factors can interfere with the expression of glutamatergic synaptic molecules, including AMPA and NMDA receptor subunits, thereby affecting synaptic plasticity, which is strongly associated with cognitive impairment in patients with depression [68, 69]. Therefore, further detailed studies are needed to investigate the effects of PDA NPs on synaptic activity as well as the entire neural network.

Extensive studies have shown that decreased concentrations of monoamine neurotransmitters, including serotonin (5-HT), norepinephrine (NE) and dopamine (DA), are one of the main causes of depression [70]. Nanoparticles enter epithelial cells mainly by endocytosis, then by lysosomes, and finally by exocytosis into the brain [71, 72]. PDA NPs contain a large number of non-covalent supramolecular structures, which are easily destroyed by hydrogen ions in the acidic environment [73]. Therefore, we speculate that PDA NPs pass through BBB during which PDA NPs first enter the acidic lysosomal environment to be digested and decomposed, and then release their metabolites into the central nervous system by exocytosis. Additionally, PDA NPs derived from oxidative self-polymerization of DA contain 14.2% unpolymerized DA [74]. We hypothesized that the metabolites of PDA NPs in the brain might include DA monomers, thereby increasing the concentration of DA in the CNS and improving depressive symptoms. In addition, DA can be converted to NE by dopamine β-hydroxylase (DβH) [75], which also exerts antidepressant effects. In general, PDA NPs increase monoamine neurotransmitters in the CNS to play an antidepressant role, which is a reasonable hypothesis and warrants further investigation.

Nanotechnology-mediated antidepressant therapy is emerging as a new treatment strategy for depression, among which nanocarrier drug delivery system has received particular attention [58]. Recently, a photoresponsive macrophage drug-carrying system has been successfully employed to prevent inflammation-related depression in the mice, which can effectively penetrate the blood–brain barrier to improve drug bioavailability and precisely target the central M1 microglia [64]. However, such antidepressant nanomedicine usually has a complex structure with multiple components, which is relatively difficult and expensive to synthesize and clinical translation. In addition, the most important obstacle is its potential harmful residue and toxicity [76]. In this study, we developed a carrier-free drug delivery system based on the unique nanometer effect and antioxidant capacity of PDA NPs. Compared with other studies, the components of PDA NPs are relatively simple and can be prepared by the simplest one-step synthesis method. In addition, for such a carrier-free drug delivery system, we did not need to consider the cell damage caused by nanocarriers degradation [26]. Moreover, PDA NPs show good biocompatibility both in vitro and in vivo, which has been well demonstrated in many other literatures [31–33]. Nevertheless, further studies are required to examine the long-term toxicity of PDA NPs on normal tissues, which is essential for their translational clinical application.

## Conclusion

In summary, our studies explored PDA NPs had a good therapeutic effect on the inflammatory depression. According to anti-oxidative and anti-inflammatory properties as well as the unique nanometer effect, PDA NPs played a protective role, not only reversing LPS induced peripheral inflammation, but also entering the brain to act on TLR4/NF-κB signaling pathway to inhibit central nervous inflammation and restore synaptic loss. Such special effects might be an appropriate treatment strategy for depression caused by inflammatory infection. Thus, this study provided the great possibility of applying nanomedicine for inflammatory depression or other inflammatory brain disease.

## Methods

### Chemical and materials

Dopamine hydrochloride, N, N-dimethylformamide (DMF) and Cy5.5 NHS ester were purchased from Aladdin Reagents of China. Lipopolysaccharide (LPS) was purchased from Sigma-Aldrich. 2,2-Diphenyl-1-picrylhydrazyl (DPPH) was purchased from Med chem express. Dihydroethidium (DHE) was purchased from Beyotime Biotechnology. PC-12 cells, Minimum Essential Medium (MEM) and fetal bovine serum (FBS) were achieved from Procell Life Science & Technology.

### Preparation of PDA nanoparticles

Based on our pre-experimental experience, we mixed NH_4_OH (0.6 ml), ethanol (32 ml), and water (72 ml) and stir it at room temperature for 0.5 h. Dopamine hydrochloride (400 mg) was dissolved in 10 ml of deionized water, added to the above mixture followed by self-polymerization reaction for 18 h. Then, the PDA nanoparticles were purified by dialysis in deionized water using dialysis bags (molecular weight cut-off value of 8000 Da) for 5 days.

### Characterization

The morphology of PDA NPs was observed by field emission scanning electron microscopy (SEM, ZEISS Sigma 300).The chemical structure was acquired by using Fourier transform infrared spectroscopy (FTIR, Nicolet iS20, Thermo). Dynamic light scattering (DLS) and zeta potential of PDA NPs and Cy5.5-PDA-SiO_2_ NPs were measured by ZS Nano (Malvern Instruments Ltd, NANO ZS90). The core–shell structure of Cy5.5-PDA-SiO_2_ NPs were obtained by Transmission Electron Microscopy (TEM, JEM2100F).

### ROS scavenging assay

#### DPPH radical scavenging assay

First, we prepared 0.1 mM of DPPH solution in 95% ethanol, then 4 ml of DPPH solution was mixed with different doses of PDA NPs or ascorbic acid. The concentrations of PDA NPs or ascorbic acid in the mixed solution were 5, 10, 20, 40 and 50 μg/ml. Scavenging activity was assessed by monitoring the decrease in absorbance at 516 nm after 30 min in the dark. The DPPH radical scavenging activity was calculated as I = [1 − (Ai − Aj)/Ac] 100%, where Ac is the absorbance of DPPH in solution without sample, Ai is the mixture of sample and DPPH solution, and Aj is the absorbance of sample mixed with 95% ethanol.

#### Intracellular ROS scavenging assay

To evaluate the ability of PDA NPs to scavenge ROS in vitro, PC12 cells were seeded in 12-well plates and cultured at a density of 10,000 cells per well for 12 h. After removing the medium, cells were incubated with MEM containing FBS (10%) and PDA NPs (0, 40 or 80 μg/ml) for 6 h, then cells were washed with PBS to remove excess PDA NPs. After stimulation with LPS (1 mg/ml) for 30 min, the cells were rinsed with PBS again, followed by incubated with DHE at 37 °C for 0.5 h in the dark. Group without any treatment was defined as control. Fluorescence microscopy images were collected on an Olympus imaging system.

#### PC-12 cytotoxicity assay

PC-12 cells were cultured in MEM containing FBS (10%) in the 96-well plates for 24 h, with a density of 5000 cells per well. After removing the medium, cells were incubated with new medium and different concentrations of PDA NPs (1, 10, 20, 40, 80, 100 or 200 μg/ml) for 24, 48 or 72 h. Next, the original medium and PDA NPs were withdrawn, and the cells were washed twice with medium to remove the effect of PDA NPs on the absorbance. Then 90 μl of new medium and 10 μl CCK-8 reagent (C0037, Beijing Biotechnology, Shanghai, China) were added to incubation for 1 h in each hole. Finally, the optical density (OD) at 450 nm was acquired using a microplate reader (Bio-Rad Laboratories Inc.) and the cell survival rate was calculated as cell viability (%) = (A experimental group/A control group) × 100%.

### Animals

All experimental procedures were approved by the Ethics Committee of Zhengzhou University. One hundred and twenty 8-week-old male C57BL/6 J mice were purchased from the Animal Center of Zhengzhou University, Zhengzhou, China. This study was conducted in male mice to control for hormonal variables. Five animals were housed per cage in controlled temperature (24 ± 1 °C) and light period (12 h light and dark cycle) with free access to water and chow. All animal experiments were carried out in line with the National Institutes of Health guide for the care and use of Laboratory animals.

### Inflammation-induced depression model

LPS (Sigma-Aldrich, St. Louis, MO; O111:B4, 0.83 mg/kg) was diluted in normal saline and administered intraperitoneally. According to previous studies, a single dose of LPS is recognized to induce anxiety- and depression-like behaviors in mice [6, 77].

### Drug administration and animal grouping

To explore whether PDA NPs can alleviate inflammation-induced anxiety- and depression-like behaviors, mice were randomly divided into one of the following groups (N = 12/group): saline + PBS group, saline + PDA NPs group, LPS + PBS group and LPS + PDA NPs group. Mice in the LPS + PBS, the LPS + PDA NPs groups were intraperitoneally injected with 0.83 mg/kg of LPS, and the other two groups were injected with normal saline. 30 min after LPS injection, mice in the saline + PDA NPs and the LPS + PDA NPs groups were intraperitoneally injected with 10 mg/kg of PDA NPs, and the same volume of PBS was injected in the other groups. In this study, this protocol was used for virtually all in vivo experiments, including behavioral or biochemical assessments.

### Behavioral tests

A well-trained investigator blinded to grouping performed the behavioral tests in a sound-isolated room including the open field test (OFT), elevated plus maze (EPM), forced swimming test (FST), and tail suspension test (TST).

### Open field test (OFT)

Twenty-four hours following LPS injection, the spontaneous activity of the mice in the open field test was recorded using Panlab SMART 3.0 video tracking software (RWD, Shenzhen, China). Animals were placed in the center of a white experimental box (50 × 50 × 50 cm) in a room with dim light and allowed to explore freely for 5 min. The information automatically recorded was the total distance moved, time spent in and the entries into the center.

### Elevated plus-maze test (EPM)

Four hours after OFT, EPM was used to evaluate the anxiety-like behavior of mice. The maze is a plus-cross-shaped apparatus including two open arms (30 × 5 × 0.3 cm) and two enclosed arms by walls (30 × 5 × 15 cm) elevated 50 cm above the floor. Firstly, we put the mice in the open field for 5 min, then place them in the central platform facing an open arm and allowed them to explore spontaneously for 5 min. The time spent in and the entries into the open arms used as an anxiety index were automatically recorded using Panlab SMART 3.0video tracking software (RWD, Shenzhen, China). If a fall occurred, the animal was removed from the study.

### Forced swimming test (FST)

The test was performed at forty-eight hours after LPS injection to measure depression-like behavior. Mice were placed in a hyaline cylinder (20 cm diameter × 30 cm height) filled with water (23–25 °C) to a depth of 15 cm and forced to swim for 6 min. The immobile time during the last 4 min was counted by an observer blinded to the treatment conditions, which refers to the time when the mouse passively floated in the water without struggling, except those to maintain balance in the water.

At the end of each experiment, the water in the container was changed, and the mice were wiped dry with towels and transferred to their original cages.

### Tail suspension test (TST)

Mice were suspended to a hook by wrapping the tail using adhesive tape. The immobility time was considered only when they hung passively and completely motionless and quantified for the next 4 min of the total 6 min testing period.

The behavioral apparatus was sterilized with 70% ethanol between each test to prevent behavioral changes in animals due to odor use.

### Collection of blood and spleen

24 hour after LPS injection, the mice were deeply anesthetized with isoflurane (5%).

Blood was collected by cardiac puncture and stored in a 4 °C refrigerator overnight. The supernatant was collected by centrifugation at 3000 rpm for 10 min for serum cytokines analysis. Spleen was dissected from the abdomen and immediately weighed.

### Enzyme-linked immune sorbent assay (ELISA)

The serum levels of IL1β and TNFα were determined by using an ELISA kit (Elabscience, China) according to the instructions.

### In vivo imaging

Since the light-absorbing properties of PDA NPs can lead to the quenching of the surface-grafted fluorescent molecules, we introduced mesoporous silica nanoparticles (Meso-SiO_2_ NPs) as the core to physically adsorb Cy5.5 fluorescent molecules, and finally dopamine oxidative self-polymerization to form Cy5.5-Meso-SiO_2_@PDA NPs, which were injected intraperitoneally to characterize the ability of PDA NPs to cross the blood–brain barrier. After 2 and 24 h, we used an in vivo imaging system (IVIS Spectrum, PerkinElmer, USA) to observe the fluorescence intensity of Cy5.5 reflecting the entry of nanoparticles into the brain.

### Detection of superoxide dismutase (SOD) and malondialdehyde (MDA)

Superoxide dismutase (SOD) is an antioxidant enzyme that protects neural tissue from oxidative stress, while malondialdehyde (MDA) is a product of lipid peroxidation. These two indicators were measured using commercial testing kits (Beyotime Biotechnology Institute, Nantong, China) according to the manufacturers’ instructions, reflecting the potential ability of the body to resist oxidation.

### Western blot analysis

Hippocampal tissue and medial prefrontal cortex (mPFC) were carefully isolated from each mouse and then immediately homogenized by adding pre-chilled RIPA lysis buffer (CW2334, CW biotech) and phosphatase inhibitor cocktail (CW2383, CW biotech) using a grinder (KZ-II, Servicebio). The lysate was centrifuged at 14,000 g for 15 min at 4 °C to obtain the supernatant, the protein concentration of which was quantified by using the BCA protein detection kit (GK10009, GLPBIO). Equal amounts of protein samples (20 μg) were separated by 10% SDS-PAGE and transferred to nitrocellulose membranes (Millipore, Bedford, MA, USA), blocked for 1 h in TBST containing 3% nonfat milk, and incubated at 4 °C overnight with the different primary antibodies, including anti-IL1β (1.15 μg/ml, A1112, Abclonal), anti-TNFα (1 μg/ml, A11534, Abclonal), anti-CD86 (1.1 μg/ml, E5W6H, Cell-Signaling), anti-TLR4 (1.5 μg/ml, A5258, Abclonal), anti-MyD88 (1 μg/ml, 67969, Proteintech), anti-p-NF-κB (1 μg/ml, bs-5661R, Bioss), anti-GAPDH (0.06 μg/ml, 10494, Proteintech), anti-PSD95 (1 μg/ml, AB18258, Abcam), anti-vGLUT1 (0.5 μg/ml, 135011, SYSY) and anti-β-actin (1 μg/ml, 66009, Proteintech). After washed with Tris Buffer Saline with Tween-20 (TBST) (T1085, Solarbio) three times, the membranes were incubated for 2 h with second antibodies at room temperature. Secondary antibodies were used as follows: Goat anti-Rabbit IgG-HRP (0.2 μg/ml, SA00001-2, Proteintech) or Goat anti-mouse IgG-HRP (0.2 μg/ml, SA00001-1, Proteintech). The Immunoreactivity bands were detected by enhanced chemiluminescence (WBKLS0100, Millipore) and analyzed with Image J software (National Institutes of Health, Bethesda, MD, USA).

### Immunohistochemistry

Mice were deeply anesthetized with isoflurane and slowly perfused with phosphate-buffered saline (PBS) and 4% paraformaldehyde (PFA). Brains were carefully removed and postfixed in 4% PFA overnight, then dehydrated with 20% and 30% sugar in PBS and coronally cut into 25 µm-thick brain slices using a cryostat (CM1950, Leica). The sections were blocked with 5% normal goat serum and 0.3% Triton X-100 in PBS for 1 h at room temperature followed by incubation with primary antibody against Iba1 (1 μg/ml, 019-19741, Wako) or 8-OHDG (5 μg/ml, AB62623, Abcam) overnight at 4 °C. Next, wash the sections three times with PBS before incubating them with the goat anti-rabbit IgG-Cy3 (3.75 μg/ml, 115165045, Jackson ImmunoResearch) or goat anti-mouse IgG-FITC (15 μg/ml, 115095146, Jackson ImmunoResearch) for 2 h at room temperature. Finally, 10 μg/ml 4′,6-diamidino-2-phenyl-indole (DAPI) (C0065, Solarbio) immersed the slices for 5 min for nuclear staining for localization. Fluorescence microscopy images were collected by a confocal scanning microscope (A1 MP +, Nikon).

### Golgi staining

With reference to our previous research, Golgi staining can be used to detect the form and dendritic spines of neurons in the brain [78].

According to the instructions of Golgi Stain Kit (#PK401, FD Neuroethologies, Columbia, MD, USA), firstly, we prepared an equal amount of mixed A and B solutions 24 h in advance, and then immediately immersed the dissected mice brain in it in the dark at room temperature for 2–3 weeks. Next, the brain tissue was transferred to the solution C for 3 days. Then, the tissue was cut into 100 μm-thick brain slices with a vibrating microtome (VT1000; Leica Microsystems, Germany) and mounted on gelatin-coated slides for staining. After dehydration and xylene clearing, the slides were embedded in neutral balsam. Images were taken by using a confocal microscope at X20 and X100 magnification (Olympus FluoView FV1000, Tokyo, Japan).

### Hemolysis assay

Red blood cells were separated from serum, and diluted to 2% with PBS. Diluted red blood cells (1 ml) were mixed with PBS (1 ml) as negative control, 0.2% Triton X-100 (1 ml) as positive control, and 200 μg/ml PDA NPs (1 ml) as experimental samples. After incubation at 37 °C for 4 h and centrifugation at 1000 rpm for 15 min, the absorbance of the supernatants at 545 nm was measured on a Bio-Rad microplate reader.

### Hematoxylin–eosin (H&E) staining

The major organs of mice (heart, liver, spleen, lung, kidney) were fixed with 4% PFA, embedded in paraffin. The samples were sectioned with a microtome (RM2016, Shanghai Leica Instrument) at the thickness of 4 mm after dehydration in an alcohol gradient. Subsequently, we performed H&E staining and observed the pathological changes of major organs under a light microscope (200 ×).

### Statistical analysis

GraphPad Prism software (version 8) were used for statistical analysis and drawing plots. Data in figures were presented as the mean ± SEM. The difference between groups were evaluated by one-way analysis of variance (ANOVA) followed by Bonferroni test for post hoc comparisons. Statistical significance was calculated as *P < 0.05, **P < 0.01.

## Supplementary Information

**Additional file 1: Figure S1. **Medium dose of PDA NPs ameliorated LPS-induced anxiety- and depression-like behaviors. Figure S2. Preparation and characterization of Cy5.5-PDA-SiO2 NPs. Figure S3. PDA NPs reversed the increased microglial numbers induced by LPS in the hippocampus. Figure S4. PDA NPs restrained the pro-inflammatory transformation of microglia induced by LPS in the mPFC. Figure S5. PDA NPs alleviated the level of oxidative stress induced by LPS. Figure S6. PDA NPs play an anti-inflammatory and antidepressant role through TLR4/NF-κB signaling pathway. Figure S7. Typical SEM images of silica (SiO2) NPs (scale bar: 1 μm). Figure S8. PDA NPs play an anti-inflammatory and antidepressant role through TLR4/NF-κB signaling pathway. Figure S9. PDA NPs alleviated the impairment of synaptic structures in the mPFC. Figure S10. The cell viability of PC-12 cells after treatment for 48 h or 72 h with the different concentrations of PDA NPs.

## Data Availability

All data needed to evaluate the conclusions in this study are present in this article and additional materials. Additional data related to this paper may be requested from the authors.
