# Supplementary material for: Melanin-like polydopamine nanoparticles mediating anti-inflammatory and rescuing synaptic loss for inflammatory depression therapy
Source: J Nanobiotechnology. 2023 Feb 10;21:52. doi: 10.1186/s12951-023-01807-4 (PMC9913011; doi:10.1186/s12951-023-01807-4)
Supplement: Supplementary file 1 — Additional file 1: Figure S1. Medium dose of PDA NPs ameliorated LPS-induced anxiety- and depression-like behaviors. Figure S2. Preparation and characterization of Cy5.5-PDA-SiO2 NPs. Figure S3. PDA NPs reversed the increased microglial numbers induced by LPS in the hippocampus. Figure S4. PDA NPs restrained the pro-inflammatory transformation of microglia induced by LPS in the mPFC. Figure S5. PDA NPs alleviated the level of oxidative stress induced by LPS. Figure S6. PDA NPs play an anti-inflammatory and antidepressant role through TLR4/NF-κB signaling pathway. Figure S7. Typical SEM images of silica (SiO2) NPs (scale bar: 1 μm). Figure S8. PDA NPs play an anti-inflammatory and antidepressant role through TLR4/NF-κB signaling pathway. Figure S9. PDA NPs alleviated the impairment of synaptic structures in the mPFC. Figure S10. The cell viability of PC-12 cells after treatment for 48 h or 72 h with the different concentrations of PDA NPs. [file 12951_2023_1807_MOESM1_ESM.docx]

**Supporting information**

Melanin-like polydopamine nanoparticles mediating anti-inflammatory and rescuing synaptic loss for inflammatory depression therapy

Ting-ting Zhu^1,2^, He Wang^1^, Han-wen Gu^1,2^, Ling-sha Ju^1^, Xin-miao Wu^1,2^, Wei-tong Pan^1,2^, Ming-ming Zhao^1,2^, Jian-jun Yang^1,2*^, Pan-miao Liu^1,2*^


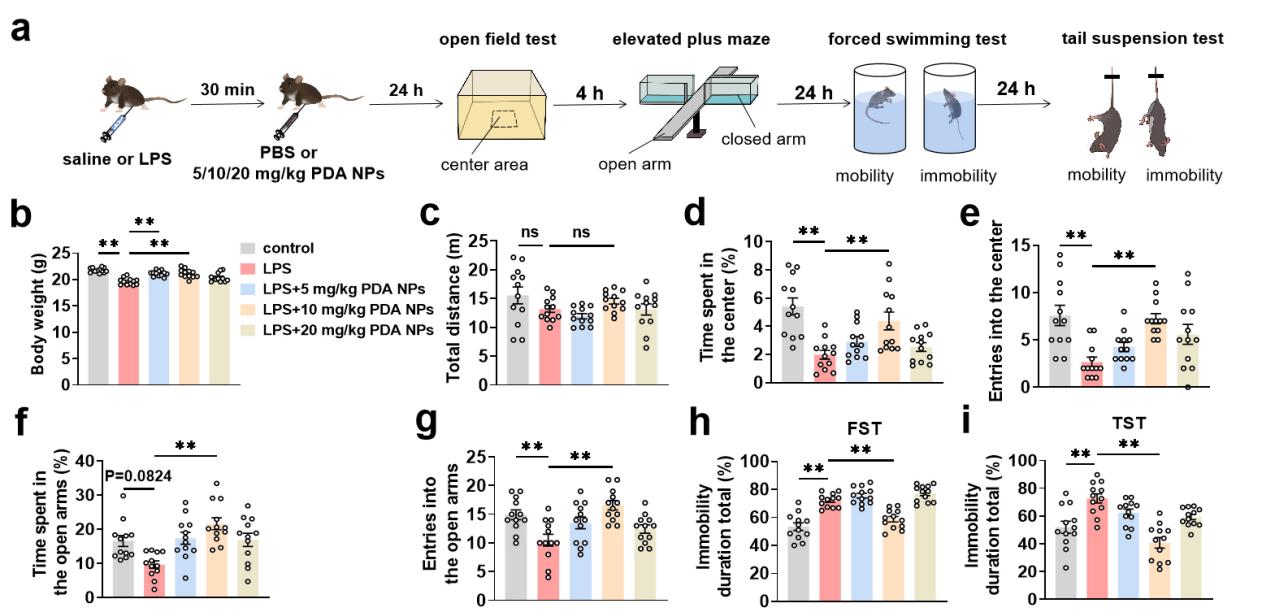


**Figure S1. Medium dose of PDA NPs ameliorated LPS-induced anxiety- and depression-like behaviors.** (**a**) Schematic illustration of the experimental procedure. PDA NPs (5/10/20 mg kg^−1^) were intraperitoneally injected at 30 min after LPS injection, and then the therapeutic effect was evaluated by anxiety and depression behavior test, including open field test (OFT), elevated plus maze (EPM), forced swimming test (FST) and tail suspension test (TST). (**b**) Body weight of mice in the five groups at 48 hours after LPS treatment (F (4, 55) = 17.98, *P* < 0.0001). Bar graphs with dots showing (**c**) total distances as well as percentage of (**d**) time spent in and (**e**) entries into the center of the arena during the 5 min in the OFT (Distances: F (4, 55) = 2.537, *P* = 0.0502; Time: F (4, 55) = 9.317, *P* < 0.0001; Entries: F (4, 55) = 6.993, *P* < 0.0001). Bar graphs with dots showing percentage of (**f**) time spent in and (**g**) entries into the open arms of the maze (Time: F (4, 55) = 6.836, *P* = 0.0002; Entries: F (4, 55) = 6.783, *P* = 0.0002). (**h**) Bar graphs with dots showing percentage of immobility time in FST and (**i**) TST (FST: F (4, 55) = 30.14, *P* < 0.0001; TST: F (4, 55) = 12.86, *P* < 0.0001). Data are presented as the means ± SEM (n = 12 in each group). Results were analyzed by one-way analysis of variance (ANOVA) followed by Bonferroni test for post hoc comparisons. (*): *p* < 0.05, (**): *p* < 0.01 versus indicated groups; (n.s.): not significant. **Abbreviations:** LPS: lipopolysaccharide; PBS: phosphate-buffered saline; PDA NPs: polydopamine nanoparticles; FST: forced swimming test; TST: tail suspension test.

*
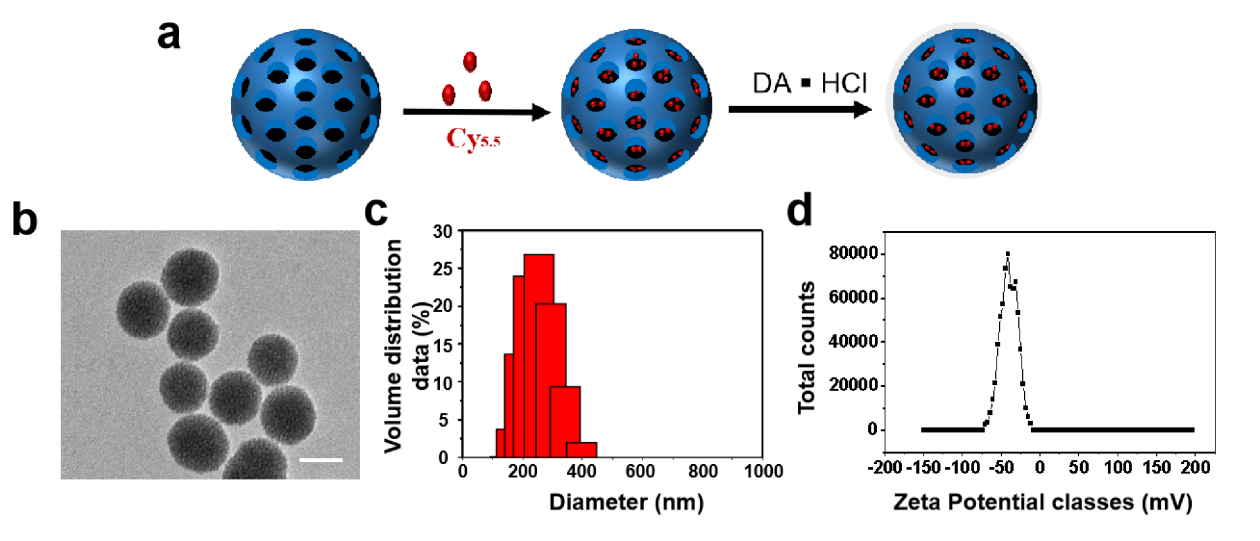
*

**Figure S2. Preparation and characterization of Cy5.5-PDA-SiO_2_ NPs.** (**a**) Schematic illustration for the synthesis of Cy5.5-PDA-SiO_2_ NPs. (**b**) TEM images of Cy5.5-PDA-SiO_2_ NPs. (scale bar = 200 nm). (**c**) DLS analysis and (**d**) Zeta potentials analysis Cy5.5-PDA-SiO_2_ NPs. **Abbreviations:** DA-HCl: dopamine hydrochloride; Cy5.5-PDA-SiO_2_ NPs: cyanine 5.5-polydopamine-silica nanoparticles; TEM: transmission electron microscopy; DLS: dynamic light scattering.


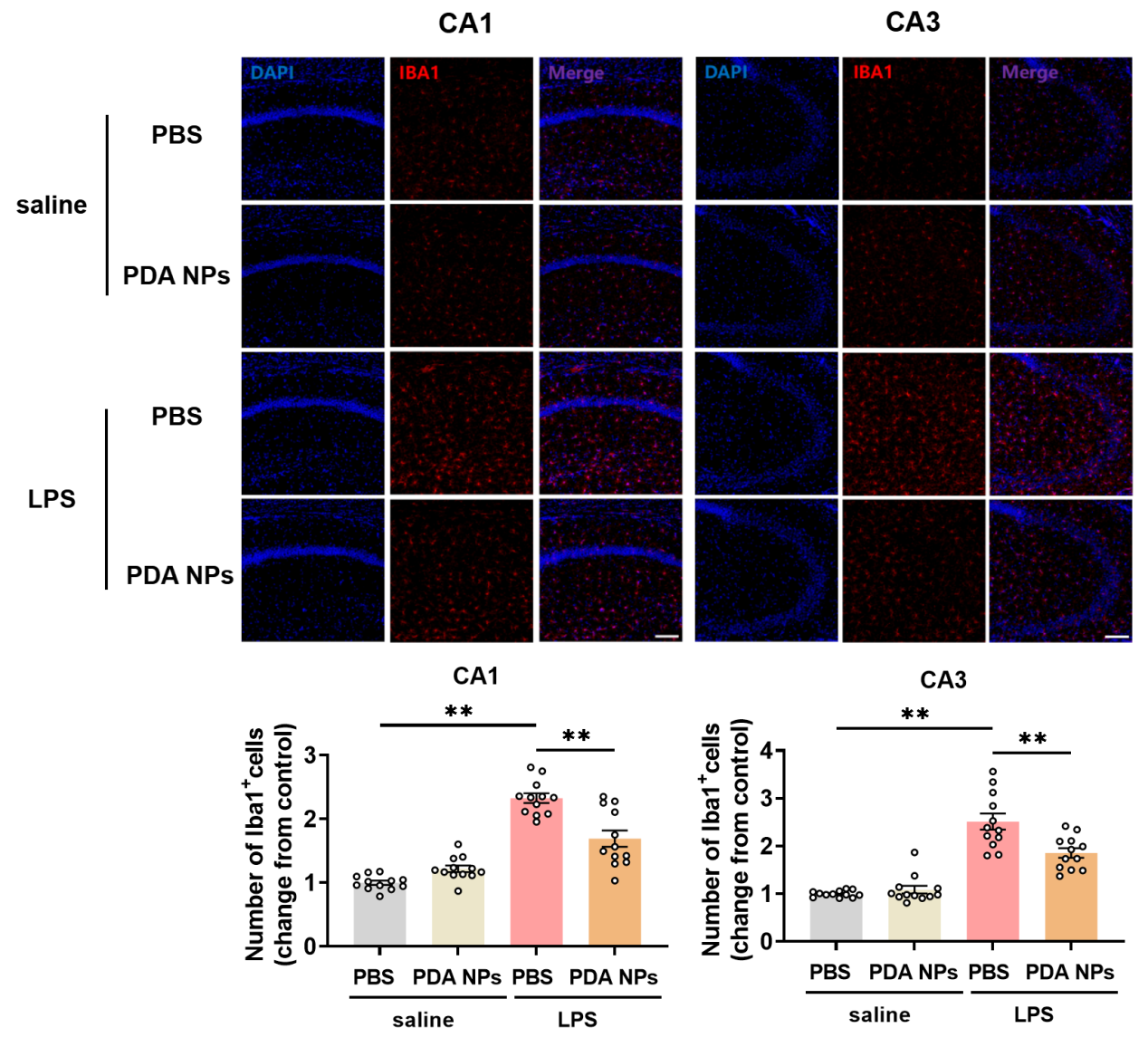


**Figure S3. PDA NPs reversed the increased microglial numbers induced by LPS in the hippocampus.** Representative immunofluorescence images and the corresponding bar graphs with dots showing Iba1^+^ microglia (red) in the CA1 (F (3, 44) = 52.29, *P* < 0.0001) and CA3 (F (3, 44) = 44.78, *P* < 0.0001) of hippocampus (12 images from 3 mice in each group). Scale bar = 100 μm. Data are presented as the means ± SEM. Results were analyzed by one-way analysis of variance (ANOVA) followed by Bonferroni test for post hoc comparisons. (*): *p* < 0.05, (**): *p* < 0.01 versus indicated groups. **Abbreviations:** LPS: lipopolysaccharide; PBS: phosphate-buffered saline; PDA NPs: polydopamine nanoparticles; Iba1: ionized calcium binding adaptor molecule 1; CA1: hippocampal CA1; CA3: hippocampal CA3.


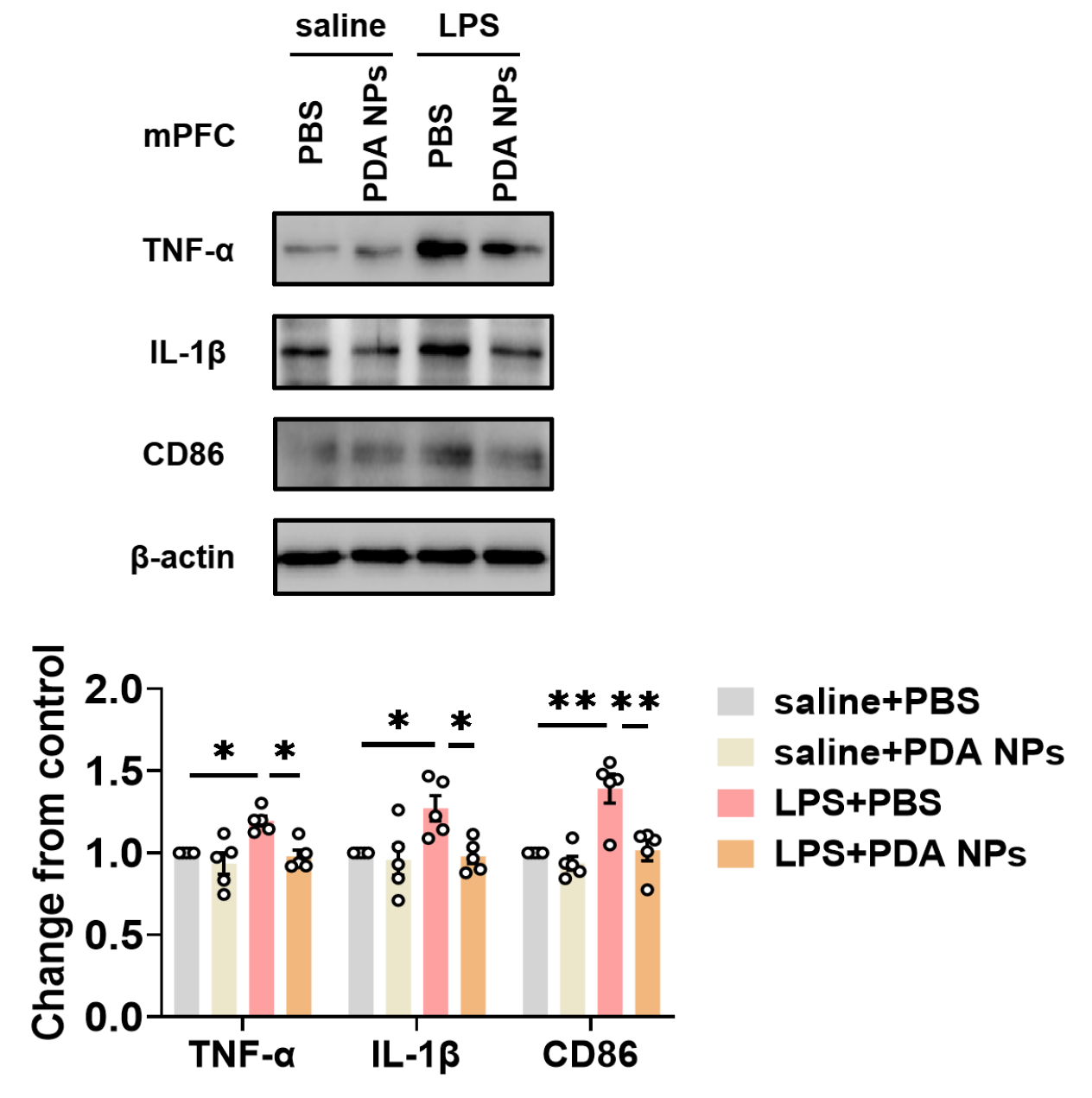


**Figure S4. PDA NPs restrained the pro-inflammatory transformation of microglia induced by LPS in the mPFC.** Representative western blot and the corresponding bar graphs with dots showing TNF-α, IL-1β and CD86 expression levels in the mPFC (TNF-α: F (3, 16) = 7.965, *P* = 0.0018; IL-1β: F (3, 16) = 5.333, *P* = 0.0097; CD86: F (3, 16) = 12.77, *P* = 0.0002) (n = 5 / group). Data are presented as the means ± SEM. Results were analyzed by one-way analysis of variance (ANOVA) followed by Bonferroni test for post hoc comparisons. (*): *p* < 0.05, (**): *p* < 0.01 versus indicated groups. **Abbreviations**: LPS: lipopolysaccharide; PBS: phosphate-buffered saline; PDA NPs: polydopamine nanoparticles; mPFC: medial prefrontal cortex; IL1β: interleukin-1 beta; TNFα: tumor necrosis factor alpha; CD86: cluster of differentiation 86.


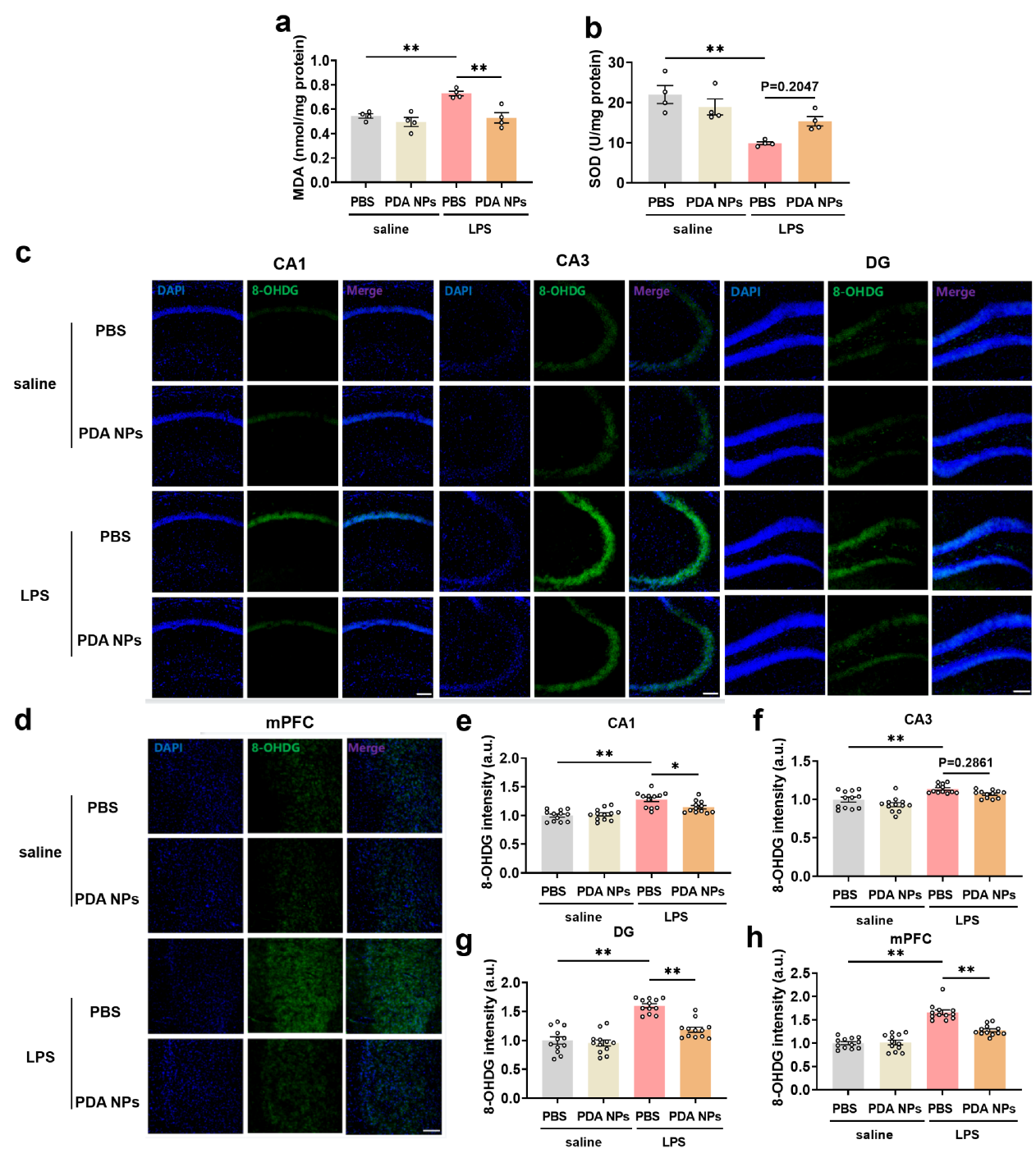


**Figure S5. PDA NPs alleviated the level of oxidative stress induced by LPS.** Quantification of (**a**) MDA content (F (3, 12) = 11.37, *P* = 0.0008) and (**b**) SOD activity (F (3, 12) = 10.30, *P*=0.0012). (**c**) Representative immunofluorescence images and the corresponding bar graphs with dots showing 8-OHDG (green) in the (**e**) CA1 (F (3, 44) = 17.07, *P* < 0.0001), (**f**) CA3 (F (3, 44) = 14.09, *P* < 0.0001) and (**g**) DG (F (3, 44) = 34.82, *P* < 0.0001) of hippocampus (12 images from 3 mice in each group). Scale bar = 100 μm. (**d**) Representative immunofluorescence images and the corresponding bar graphs with dots showing 8-OHDG (green) expression level in the (**h**) mPFC (F (3, 44) = 51.93, *P* < 0.0001) (12 images from 3 mice in each group). Scale bar = 100 μm. Data are presented as the means ± SEM. Results were analyzed by one-way analysis of variance (ANOVA) followed by Bonferroni test for post hoc comparisons. (*): *p* < 0.05, (**): *p* < 0.01 versus indicated groups. **Abbreviations**: LPS: lipopolysaccharide; PBS: phosphate-buffered saline; PDA NPs: polydopamine nanoparticles; SOD: superoxide dismutase; MDA: malondialdehyde; CA1: hippocampal CA1; CA3: hippocampal CA3; DG: dentate gyrus of hippocampus; mPFC: medial prefrontal cortex; 8-OHDG: 8-hydroxydeoxyguanosine.


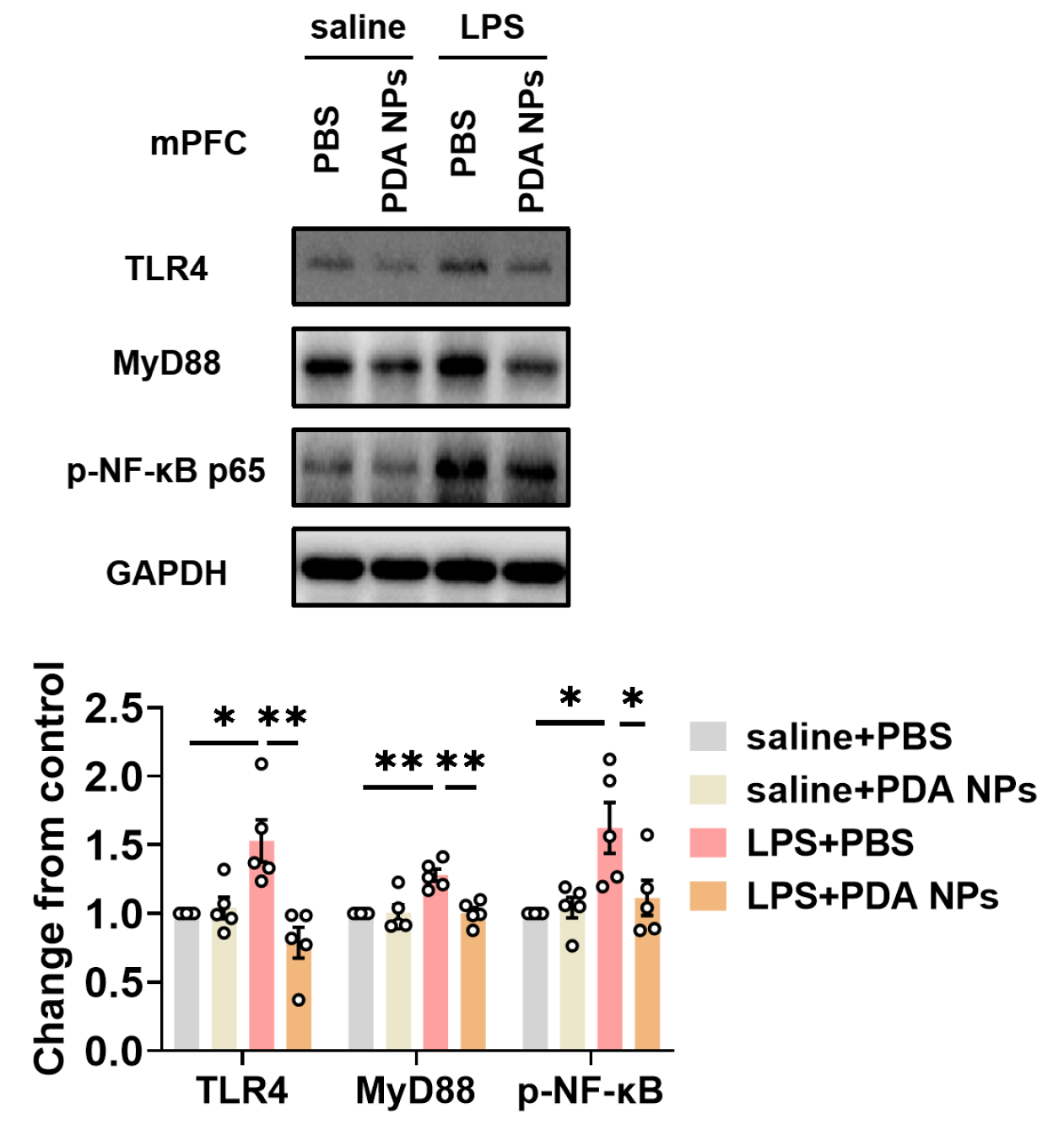


**Figure S6. PDA NPs play an anti-inflammatory and antidepressant role through TLR4/NF-κB signaling pathway.** Representative western blot and the corresponding bar graphs with dots showing TLR4, MyD88 and p-NF-κB p65 expression levels in the mPFC (TLR4: F (3, 16) = 9.262, *P* = 0.0009; MyD88: F (3, 16) = 10.83, *P* = 0.0004; p-NF-κB p65: F (3, 16) = 5.980, *P* = 0.0062). Data are presented as the means ± SEM (n = 5 / group). Results were analyzed by one-way analysis of variance (ANOVA) followed by Bonferroni test for post hoc comparisons. (*): *p* < 0.05, (**): *p* < 0.01 versus indicated groups. **Abbreviations**: LPS: lipopolysaccharide; PBS: phosphate-buffered saline; PDA NPs：polydopamine nanoparticles; mPFC: medial prefrontal cortex; TLR4: toll Like receptor 4; MyD88: myeloid differentiation factor 88; p-NF-κB: phospho-nuclear factor kappa-B.


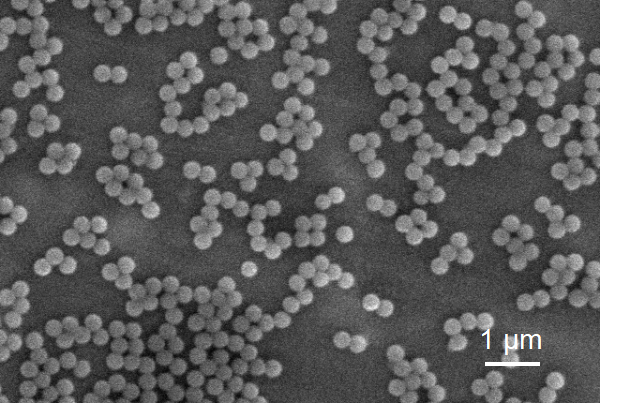


**Figure S7. Typical SEM images of silica(SiO_2_) NPs (scale bar: 1 μm).**


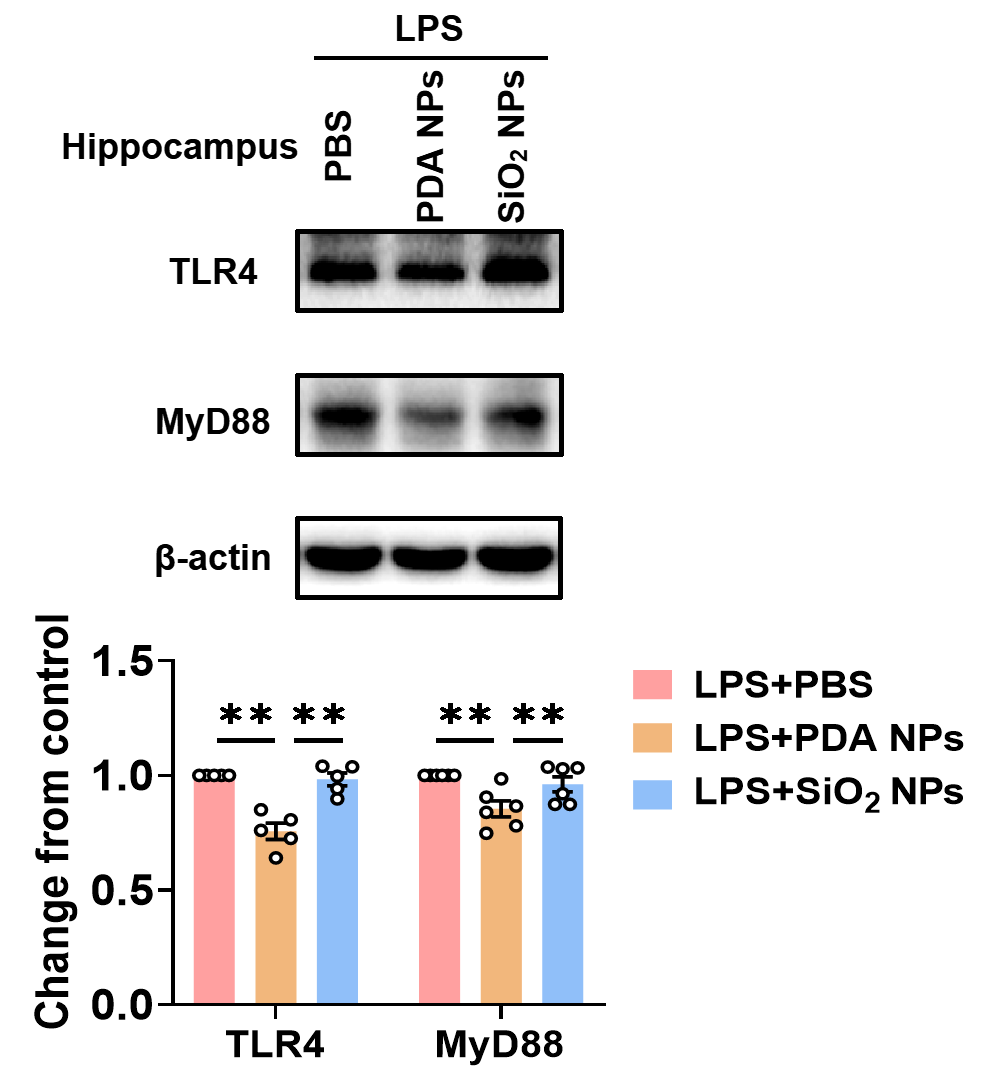


**Figure S8. PDA NPs play an anti-inflammatory and antidepressant role through TLR4/NF-κB signaling pathway.** Representative western blot and the corresponding bar graphs with dots showing hippocampal TLR4, MyD88 expression levels in LPS+PBS, LPS+PDA NPs and LPS+SiO_2_ NPs mice (TLR4: F (2, 12) = 26.98, *P* < 0.0001; MyD88: F (2, 15) = 7.437, *P* = 0.0057). Data are presented as the means ± SEM (n = 5-6 / group). Results were analyzed by one-way analysis of variance (ANOVA) followed by Bonferroni test for post hoc comparisons. (*): *p* < 0.05, (**): *p* < 0.01 versus indicated groups. **Abbreviations**: LPS: lipopolysaccharide; PBS: phosphate-buffered saline; PDA NPs：polydopamine nanoparticles; TLR4: toll Like receptor 4; MyD88: myeloid differentiation factor 88; SiO_2_ NPs: silica nanoparticles.


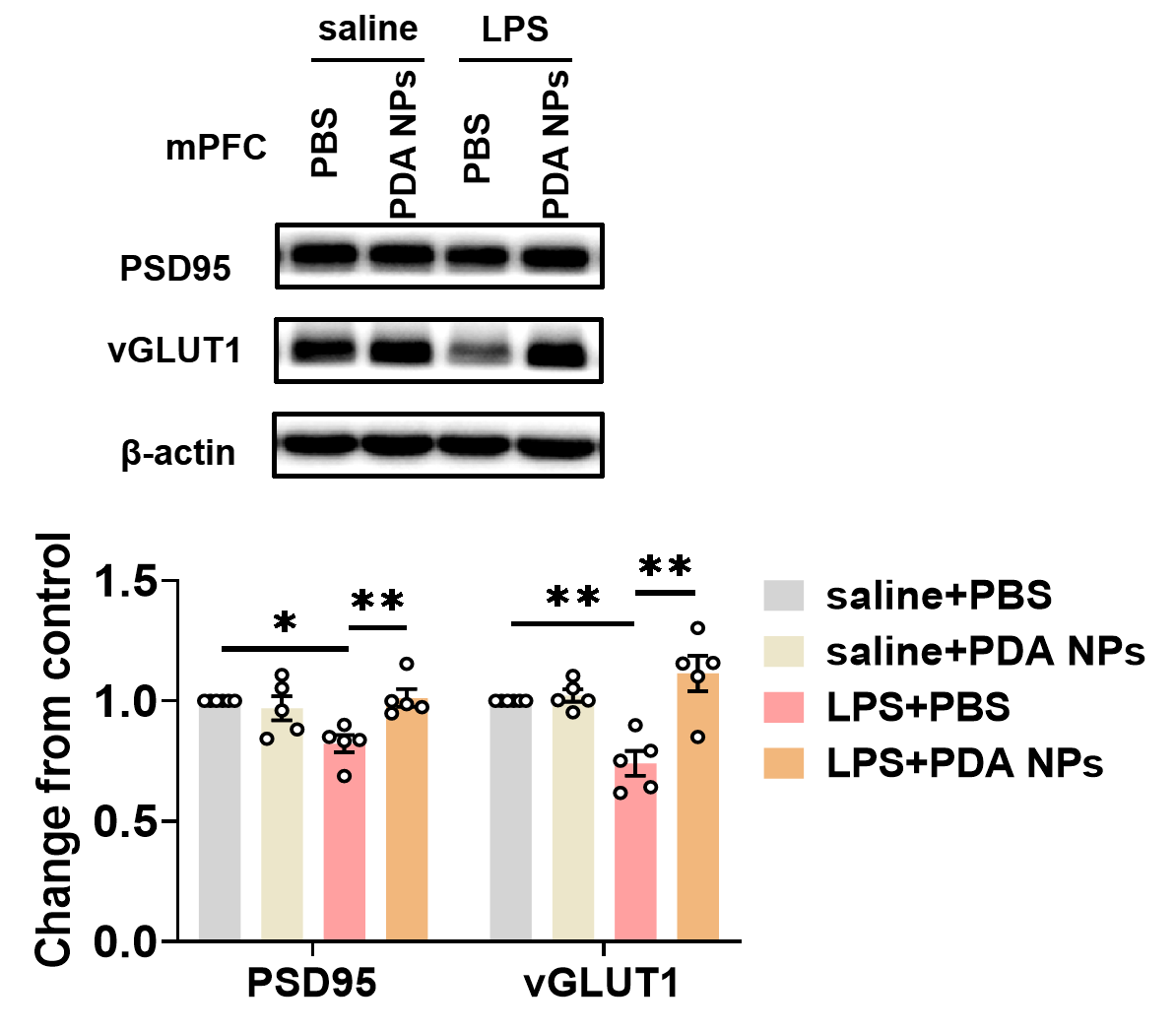


**Figure S9.** **PDA NPs alleviated the impairment of synaptic structures in the mPFC.** Representative western blot and the corresponding bar graphs with dots showing PSD-95 (F (3, 16) = 6.063, *P* = 0.0057) and vGLUT1 (F (3, 16) = 11.71, *P* = 0.0003) expression levels in the mPFC. Data are presented as the means ± SEM (n = 5 / group). Results were analyzed by one-way analysis of variance (ANOVA) followed by Bonferroni test for post hoc comparisons. (*): *p* < 0.05, (**): *p* < 0.01 versus indicated groups. **Abbreviations**: LPS: lipopolysaccharide; PBS: phosphate-buffered saline; PDA NPs: polydopamine nanoparticles; mPFC: medial prefrontal cortex; PSD95: postsynaptic density protein 95; vGLUT1: vesicular glutamate transporter 1.


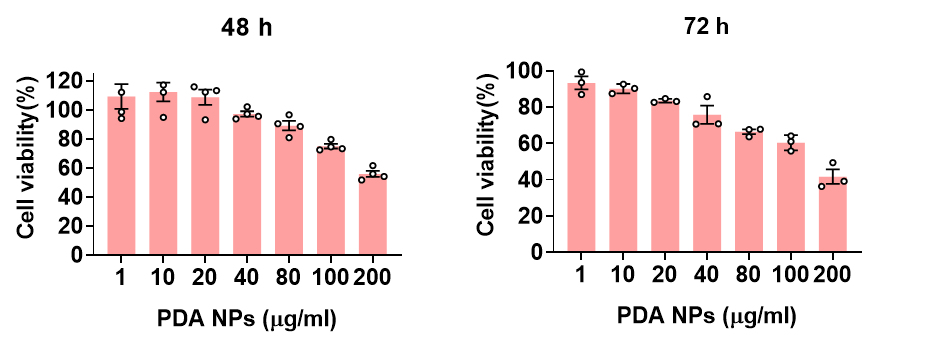


Figure S10. The cell viability of PC-12 cells after treatment for 48 h or 72 h with the different concentrations of PDA NPs.
